# Colloidal Ternary Telluride Quantum Dots for Tunable Phase Change Optics in the Visible and Near-Infrared

**DOI:** 10.1021/acsnano.3c01187

**Published:** 2023-03-27

**Authors:** Dhananjeya Kumaar, Matthias Can, Kevin Portner, Helena Weigand, Olesya Yarema, Simon Wintersteller, Florian Schenk, Darijan Boskovic, Nathan Pharizat, Robin Meinert, Evgeniia Gilshtein, Yaroslav Romanyuk, Artemios Karvounis, Rachel Grange, Alexandros Emboras, Vanessa Wood, Maksym Yarema

**Affiliations:** † Chemistry and Materials Design, Institute for Electronics, Department of Information Technology and Electrical Engineering, ETH Zürich, 8092 Zürich, Switzerland; ‡ Integrated Systems Laboratory, Department of Information Technology and Electrical Engineering, ETH Zürich, 8092 Zürich, Switzerland; § Optical Nanomaterial Group, Institute for Quantum Electronics, Department of Physics, ETH Zürich, 8093 Zürich, Switzerland; ∥ Materials and Device Engineering, Institute for Electronics, Department of Information Technology and Electrical Engineering, ETH Zürich, 8092 Zürich, Switzerland; ⊥ Laboratory for Thin Films and Photovoltaics, Empa − Swiss Federal Laboratories for Materials Science and Technology, 8600 Dübendorf, Switzerland

**Keywords:** nanoparticles, chalcogenides, amorphous structure, crystallization, reflectivity, phase-change applications, nonvolatile devices

## Abstract

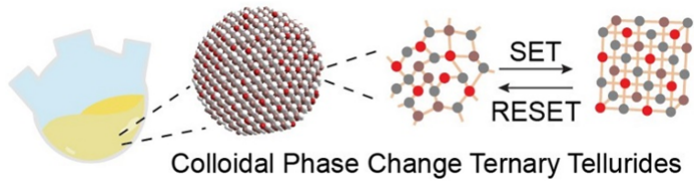

A structural change between amorphous and crystalline phase provides a basis for reliable and modular photonic and electronic devices, such as nonvolatile memory, beam steerers, solid-state reflective displays, or mid-IR antennas. In this paper, we leverage the benefits of liquid-based synthesis to access phase-change memory tellurides in the form of colloidally stable quantum dots. We report a library of ternary M_*x*_Ge_1–*x*_Te colloids (where M is Sn, Bi, Pb, In, Co, Ag) and then showcase the phase, composition, and size tunability for Sn–Ge–Te quantum dots. Full chemical control of Sn–Ge–Te quantum dots permits a systematic study of structural and optical properties of this phase-change nanomaterial. Specifically, we report composition-dependent crystallization temperature for Sn–Ge–Te quantum dots, which is notably higher compared to bulk thin films. This gives the synergistic benefit of tailoring dopant and material dimension to combine the superior aging properties and ultrafast crystallization kinetics of bulk Sn–Ge–Te, while improving memory data retention due to nanoscale size effects. Furthermore, we discover a large reflectivity contrast between amorphous and crystalline Sn–Ge–Te thin films, exceeding 0.7 in the near-IR spectrum region. We utilize these excellent phase-change optical properties of Sn–Ge–Te quantum dots along with liquid-based processability for nonvolatile multicolor images and electro-optical phase-change devices. Our colloidal approach for phase-change applications offers higher customizability of materials, simpler fabrication, and further miniaturization to the sub-10 nm phase-change devices.

Phase-change materials (PCMs) have gained major interest and revival since their use for optical data storage discs and, more recently, in storage-class memory chips. This is due to the stark contrast in material properties, such as refractive index and electrical conductivity, between the amorphous and crystalline states of PCMs. The reversible switching between the two structural states can be induced through voltage pulses (electrical switching) or laser pulses (optical switching). This results in ultrafast sub-10 ns switching rates,^1^ which in combination with high cyclability on the order of 10^8^–10^9^ makes PCM memory a competitive candidate for mainstream solid-state drives (SSDs).^2,3^ Beyond memory applications, PCMs are in high demand for tunable photonic applications, including nonvolatile reflective displays, beam steering, broadband nonvolatile optical switches, photonic tensor cores for computing, and mid-IR applications.^4−9^

The most studied PCMs fall within the pseudobinary tie line of GeTe and Sb_2_Te_3_. Aside from Sb, doping GeTe with other p-block and transitional metals has been shown to improve power efficiency, induce faster crystallization, and enhance phase-contrast during switching.^10−12^ For example, introducing Pb and Bi dopants into GeTe improves the thermoelectric properties by tuning the lattice thermal conductivity and carrier concentration, which in turn leads to improved power efficiency in phase-change memory devices.^10^ Furthermore, doping GeTe with isovalent Sn provides several immediate advantages. Sn–Ge–Te PCMs exhibit lower structural drift and faster crystallization kinetics, enabling reliable data retention at rapid switching rates.^11^ The superior properties of Sn–Ge–Te are associated with the improved structural stability of Sn coordination, which suppresses structural relaxation upon aging.^12^ Promisingly, recent theoretical studies have proposed dozens of PCM tellurides with improved properties enabling multibit data storage, sub-nm crystallization, and a number of more earth-abundant PCM compositions.^13,14^ Testing these materials experimentally requires high-throughput synthesis and device fabrication.

Wet chemistry has shown great versatility for the synthesis of inorganic nanomaterials followed by cost-effective liquid-based device fabrication methods. Colloidal synthesis can produce ultrasmall sub-10 nm semiconductor materials, called quantum dots (QDs), with size-dependent optical properties.^15^ Multiple studies have already implemented colloidal QDs in optoelectronic devices such as QLED displays, solar cells, lasers, photodetectors, or thin film transistors.^16−19^ However, little progress has been shown so far for QD-based phase-change memory.^16^ One reason is the lack of standardized synthesis protocols for PCM QDs with complex compositions, as only prototypical GeTe PCM with uniform size remains available in the form of colloidally stable QDs.^20−24^

Synthesis of multicomponent chalcogenide QDs involves many challenges, such as introducing multiple metal precursors requires balancing complex kinetics, which affects the dispersity, yield, size, and phase of QDs.^25,26^ For telluride QDs, the fast reaction kinetics of Te precursors as well as the air and moisture sensitivity of the reactants and products add to the complexity of colloidal synthesis. In this context, amide-promoted synthesis is a promising method for multicomponent telluride QDs.^27^ The key difference to other approaches is the addition of amide superbase (*e.g.*, LiN(SiMe_3_)_2_) along with the chalcogen precursor upon the hot injection step. This leads to accelerated reaction rates for metal precursors and eventually shorter reaction times and ultrasmall nanocrystal sizes. Amide-promoted synthesis is particularly effective for ternary chalcogenides because the boosting nucleation rate diminishes the reactivity difference for metal precursors in the mixture, providing a predictable way to obtain size and composition control of ternary chalcogenides, while separately from each other.^28^ Previously, several ternary tellurides, such as Cu–In–Te, Ag–In–Te, and Ag–Sb–Te, have already been prepared using amide-promoted synthesis.^29,30^ In this paper, we build on our previous works and extend amide-promoted synthesis to become a truly universal method to produce multicomponent telluride QDs.

Specifically, we achieve composition control and size uniformity for a series of ternary telluride M–Ge–Te QDs (M is Sn, Pb, In, Bi, Ag, and Co), none of which has been reported before. Most importantly, however, this paper presents an all-embracing methodology for the QD-based phase-change devices: starting from the raw materials to the full-control colloidal synthesis, comprehensive characterization of PCM QDs, and the fabrication of PCM devices. Focusing on Sn–Ge–Te QDs, we synthesize nanoparticles in both amorphous and crystalline phases and explore their phase-change structural and optical properties. We find that Sn–Ge–Te QDs exhibit some outstanding characteristics, such as a reflectivity contrast of >0.7 between amorphous and crystalline phases or notably higher crystallization temperature compared to the bulk. Using solution-based deposition, we realize nonvolatile reflective images and nonvolatile electro-optical devices, offering a proof-of-concept for liquid-borne phase-change applications.

## Results and Discussion

### Ternary Telluride Colloids

**Figure 1:**
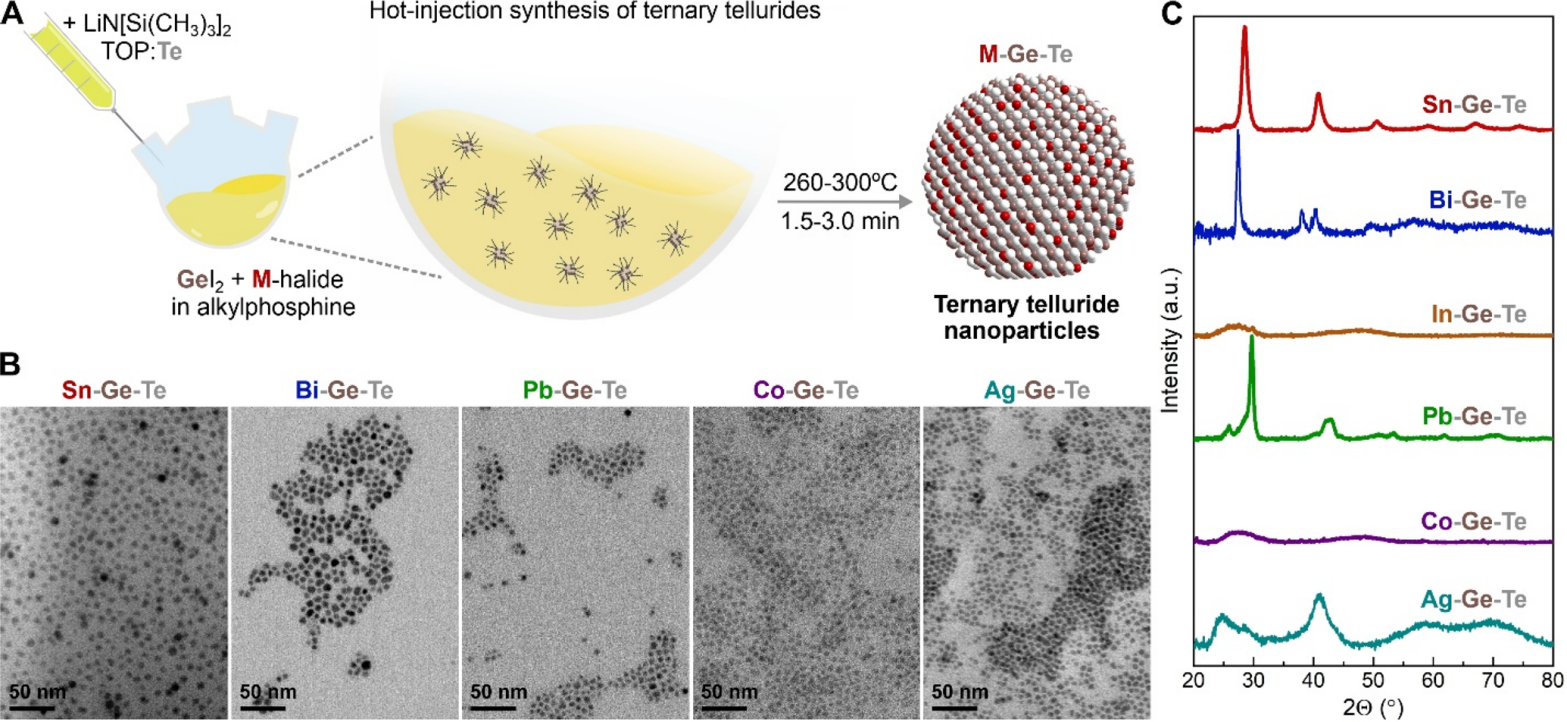
Amide-promoted synthesis of ternary telluride quantum dots. (A) Schematic illustration of the reaction. (B) Transmission electron microscopy images and (C) X-ray diffractograms of the products, forming a library of ternary telluride M–Ge-Te colloids, where M is Sn, Bi, In, Pb, Co, and Ag.

In this paper, we extend our previous work on amorphous GeTe nanoparticles^20^ to synthesize a large family of ternary telluride nanoparticles via an amide-promoted synthesis. Specifically, we add a second metal halide into the reaction flask and use the main reaction parameters (*e*.*g*., injection temperature of 250–280 °C, growth time of 1.5–3.0 min, alkylphosphine as a sole coordinating solvent) from our previous GeTe synthesis (Figure 1A and Table S1).^20^

Figure 1B shows representative TEM images for the different ternary telluride nanoparticles. The general formula for the obtained QDs can be expressed as M_*x*_Ge_1–*x*_Te (or M–Ge–Te), where M is another p-block or transition metal. We demonstrate the synthesis for Sn–Ge–Te (SGT), Bi–Ge–Te (BGT), Pb–Ge–Te (PGT), In–Ge–Te (IGT), Co–Ge–Te (CGT), and Ag–Ge–Te (AGT) in the form of monodisperse colloidal nanoparticles (Figures 1B and S1). Our QD products are typically in the sub-10 nm size range and show excellent size uniformity (Figure S2). While this work focuses on ternary telluride compositions relevant for the phase-change applications, we anticipate that other ternary M–Ge–Te nanoparticles can be prepared using our methods (Table S1). Such ternary telluride nanomaterials are often a prime choice for thermoelectric,^31^ ferroelectric,^32,33^ and infrared applications.^8,34^

Figure 1C shows X-ray diffractograms of the M–Ge–Te QDs. Ge-rich M_*x*_Ge_1–*x*_Te compositions (*i*.*e*., small *x* values) usually belong to rhombohedral α-GeTe phase (*e*.*g*., Pb–Ge–Te QDs). Decreasing the Ge content leads to the formation of other crystal structures: Sn_2_GeTe_3_ QDs exhibit closely related rock-salt SnTe phase, Bi_4_Ge_3_Te_9_ QDs show Pb_2_Bi_2_Se_5_-type structure, and Ge-rich Ag–Ge–Te QDs belong to original Ag_8_GeTe_6_ ternary phase (Figure 1C). Some of the prepared M–Ge–Te colloids have an amorphous structure, evident through the absence of Bragg reflections on XRD patterns (Figure 1C). We relate this to the higher crystallization temperatures of nanoscale M–Ge–Te, either due to the size dependence^20^ (*e*.*g*., for ultrasmall IGT QDs, Figure S1) or due to composition effects, where doping of GeTe with transition metals leads to higher crystallization (*e*.*g*., for Co_*x*_Ge_1–*x*_Te with 14 at. % of Co, Figure 1C).^35^

Finally, we exploit the benefits of amide-promoted synthesis in the independent control of the composition and size of ternary telluride QDs. While composition can be tuned proportionally to the ratio of halide precursors (Figure S3), size is regulated via the injection temperature (Figure S4). To summarize, we demonstrate the ability to fully control key properties of ternary telluride QDs, which we explore in detail for the case of Sn–Ge–Te in the following section.

### Crystalline Sn–Ge–Te Quantum Dots

**Figure 2:**
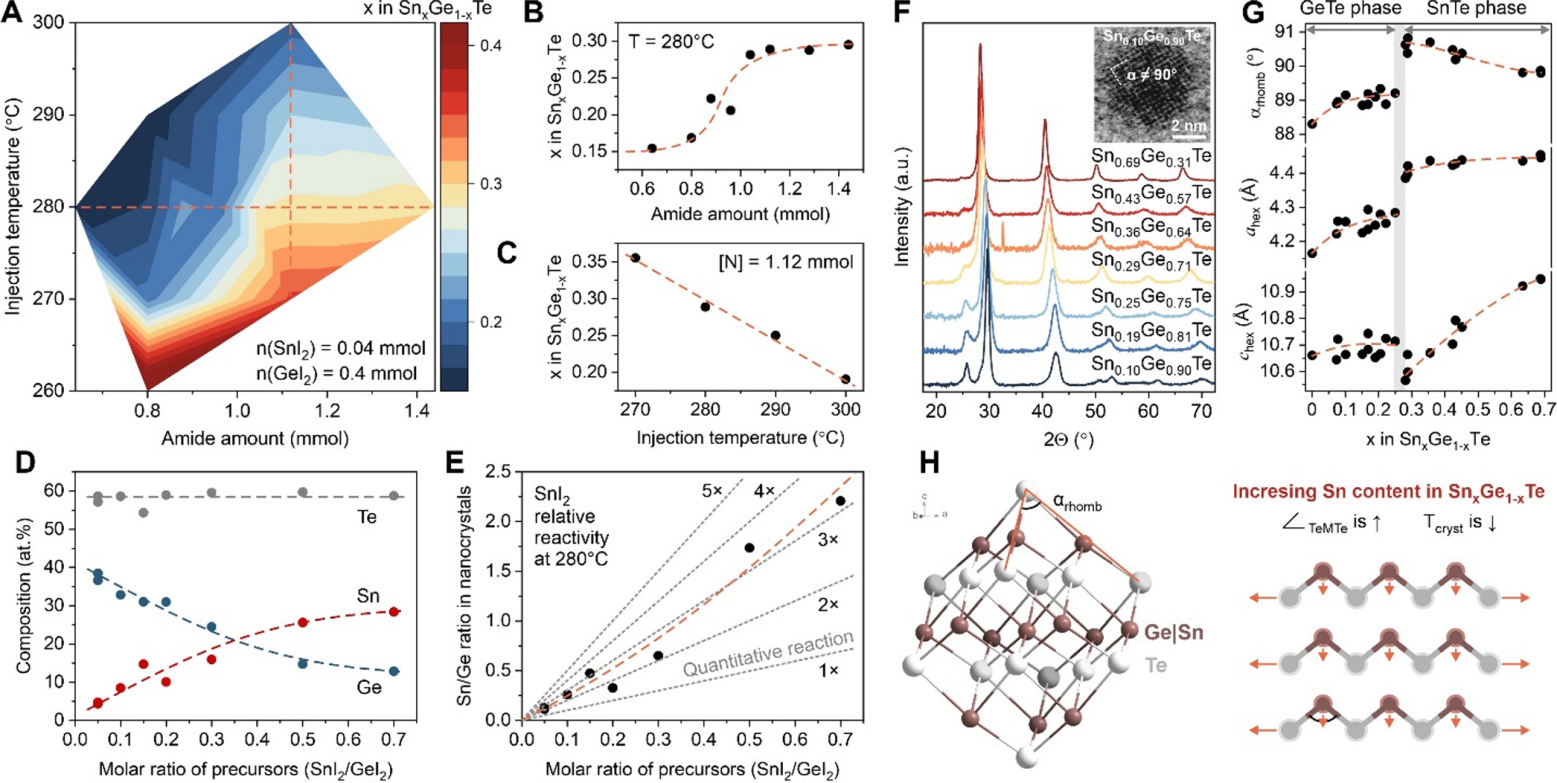
Synthesis and structure of crystalline Sn–Ge–Te (SGT) quantum dots. (A) Composition of Sn–Ge–Te quantum dots as a function of amide LiN(SiMe_3_)_2_ amount and injection temperature. (B, C) Sections of the synthesis map in (A) along the line with a constant temperature of 280 °C and constant amount of lithium amide, [N], of 1.12 mmol. (D, E) Effects of the precursor ratio on the composition of Sn–Ge–Te quantum dots and relative reactivity of metal halides. Gray dashed lines in (E) indicate the theoretical dependence for the case of equal reactivity (*i*.*e*., quantitative reaction) and higher SnI_2_ reactivity by a certain factor (*i*.*e*., 2× to 5×). (F) X-ray diffractograms of Sn_*x*_Ge_1–*x*_Te quantum dots and a high-resolution scanning transmission electron micrograph of a quantum dot, shown as an inset. (G) Crystallographic parameters of the rhombohedral lattice for Sn_*x*_Ge_1–*x*_Te quantum dots. (H) Schematic illustration of the Sn doping effects on the rhombohedral structure of GeTe.

To understand the synthetically accessible composition range for Sn–Ge–Te QDs, we perform a series of experiments, for which we systematically tune the two main reaction parameters of amide-promoted synthesis: the amount of LiN(SiMe_3_)_2_ and the injection temperature. For these experiments, we fix the molar ratio between metal precursors SnI_2_:GeI_2_ to be 1:10, keeping all other reaction conditions constant (Table S2). Figure 2A shows the experimental map as a fraction of Sn atoms in the cationic sublattice (*i.e*., *x* in the Sn_*x*_Ge_1–*x*_Te). Using this approach, we can obtain Sn_*x*_Ge_1–*x*_Te QDs with variable Sn content between 0.15 and 0.40. For all reactions, the Sn content is higher than the initial ratio of precursors, which demonstrates the higher reactivity of SnI_2_ and intermediate Sn-amide compounds in comparison to Ge precursors. We associate this with the larger size of Sn atoms, leading to weaker Sn–I and Sn–N bonds and faster conversion of Sn precursors upon nucleation and growth of Sn–Ge–Te QDs. Importantly, the amide dependence of Sn_*x*_Ge_1–*x*_Te composition is a step function with a clear plateau region above a threshold amount of approximately 1.0 mmol of LiN(SiMe_3_)_2_ (Figure 2B). Such dependency is typical for amide-promoted synthesis,^28^ offering a window of reaction conditions (*i*.*e*., excess of amide vs initial iodide ions), at which the Sn:Ge ratio in QDs remains constant and hence can be reliably controlled (Figure 2B). In agreement with previous literature on ternary selenide nanocrystals, the size of Sn_*x*_Ge_1–*x*_Te QDs (Figure S5) tends to increase around a step-like region in Figure 2B, which is related to lower ionic strength of the reaction mixture at these conditions.^28^ Finally, we observe that low amide concentrations (≤0.5 mmol) lead to much larger and ill-shaped Sn_*x*_Ge_1–*x*_Te nanoparticles due to a low number of nucleation centers.

We therefore keep the total amide amount in excess (above 1.0 mmol of LiN(SiMe_3_)_2_) for the remaining experiments. Studying the temperature effects, we observe that higher temperatures can diminish the reactivity difference between Ge and Sn precursors (Figure 2C). Overall, Ge compounds show slower reaction kinetics, as only SnTe products can be obtained when reaction temperature drops below 250 °C. Tuning the ratio of elemental precursors enables a wide composition range of Sn_*x*_Ge_1–*x*_Te QDs. Figure 2D demonstrates a monotonic and predictive change of Sn–Ge–Te composition, proportionally to the ratio of iodide salts. We note that the composition of QDs is slightly Te-rich (>50 at. % of Te), which we relate to the Te-rich surface of Sn_*x*_Ge_1–*x*_Te QDs as well as the remaining alkylphosphine-telluride molecules acting as passivating surface ligands. Figure 2E illustrates the relative reactivity of Sn and Ge precursors. While SnI_2_ is 2–3 times more reactive at these reaction conditions, the almost linear dependence suggests a co-precipitation mechanism for Sn_*x*_Ge_1–*x*_Te QD formation with no noticeable effects of parallel reactions, such as cation-exchange or separation of binary phases.

We then proceed to the structural analysis of Sn–Ge–Te QDs, using X-ray diffraction and electron microscopy (Figure 2F,G). Crystal structures of SnTe and GeTe bulk materials are similar, both having a simple-cubic-type arrangement of atoms. GeTe has a small rhombohedral distortion of the rock-salt structure, which can be quantified through the rhombohedral angle α_rhomb_ = 88.3° (α_rhomb_ = 90° for perfectly cubic rock-salt SnTe).^36^ The XRD patterns of Sn_*x*_Ge_1–*x*_Te QDs (Figure 2F) suggest a good agreement with bulk materials. Increasing the Sn content shifts Bragg reflections to shorter 2θ angles, due to the lattice expansion upon successful incorporation of larger Sn atoms into the GeTe structure. Simultaneously, Ge and Sn atoms remain randomly ordered in the cationic sublattice, evident through the absence of superstructure peaks. We notice, however, a variable rhombohedral distortion, indicated through the extent of Bragg peak splitting, for example at a 2θ range of 50–55° (Figure 2F). We also observe a small deviation from a cubic lattice visible in the high-resolution TEM image of a single Sn_*x*_Ge_1–*x*_Te QD (Figure 2F, inset).

To quantify the rhombohedral distortion, we perform a full-range Rietveld refinement XRD analysis for Sn_*x*_Ge_1–*x*_Te QDs and extract lattice parameters of the hexagonal unit cell (*a*_hex_ and *c*_hex_) in the *R*3*m* (160) space group. From the resolved lattice parameters, we can calculate the rhombohedral angle, α_rhomb_, using the following equation:
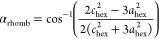


Figure 2G shows the results of the Rietveld refinement, revealing the structural details for the nanoscale GeTe–SnTe quasi-binary phase relation. In particular, we observe two phases and a small miscibility gap of a few at. % around the SnGe_3_Te_4_ composition. The existence of this miscibility gap has been hypothesized previously but lacked systematic data.^37^ We therefore provide a missing puzzle in the Sn–Ge–Te phase system with potential practical implications for thermoelectrics (*i*.*e*., secondary phase phonon scatterer),^38^ as well as ferroelectric applications (*i*.*e*., structure polarizability via atomic shifts).^33^

We can further explain the composition effects for both phases. The trigonal GeTe structure has the strongest rhombohedral distortion, *i*.*e*., the lowest α_rhomb_. Increasing SnTe content in the trigonal GeTe phase relaxes this distortion with α_rhomb_ exceeding 89° for 15–25 at. % of SnTe content (Figure 2G). The structure expands along the *a*-axis and becomes increasingly similar to the cubic rock-salt (Figure 2H). The extent of the rhombohedral distortion is of high importance for phase-change applications, increasing the structure fragility and the hybridization between s and p orbitals.^39^ We note that higher SnTe content leads to lower crystallization temperatures of phase-change tellurides,^40^ which correlates directly with the extent of rhombohedral distortion (*i*.*e*., deviation of α_rhomb_ from 90°, shown in Figure 2G). Above 28 at. % SnTe content, the Sn_*x*_Ge_1–*x*_Te material switches to oblate rhomboid (*i*.*e*., α_rhomb_ > 90°) and changes gradually to a perfect rock-salt cubic structure for SnTe-rich ternary compositions via expansion of the *c*-axis (Figure 2G).

### Amorphous Sn–Ge–Te Nanoparticles

**Figure 3:**
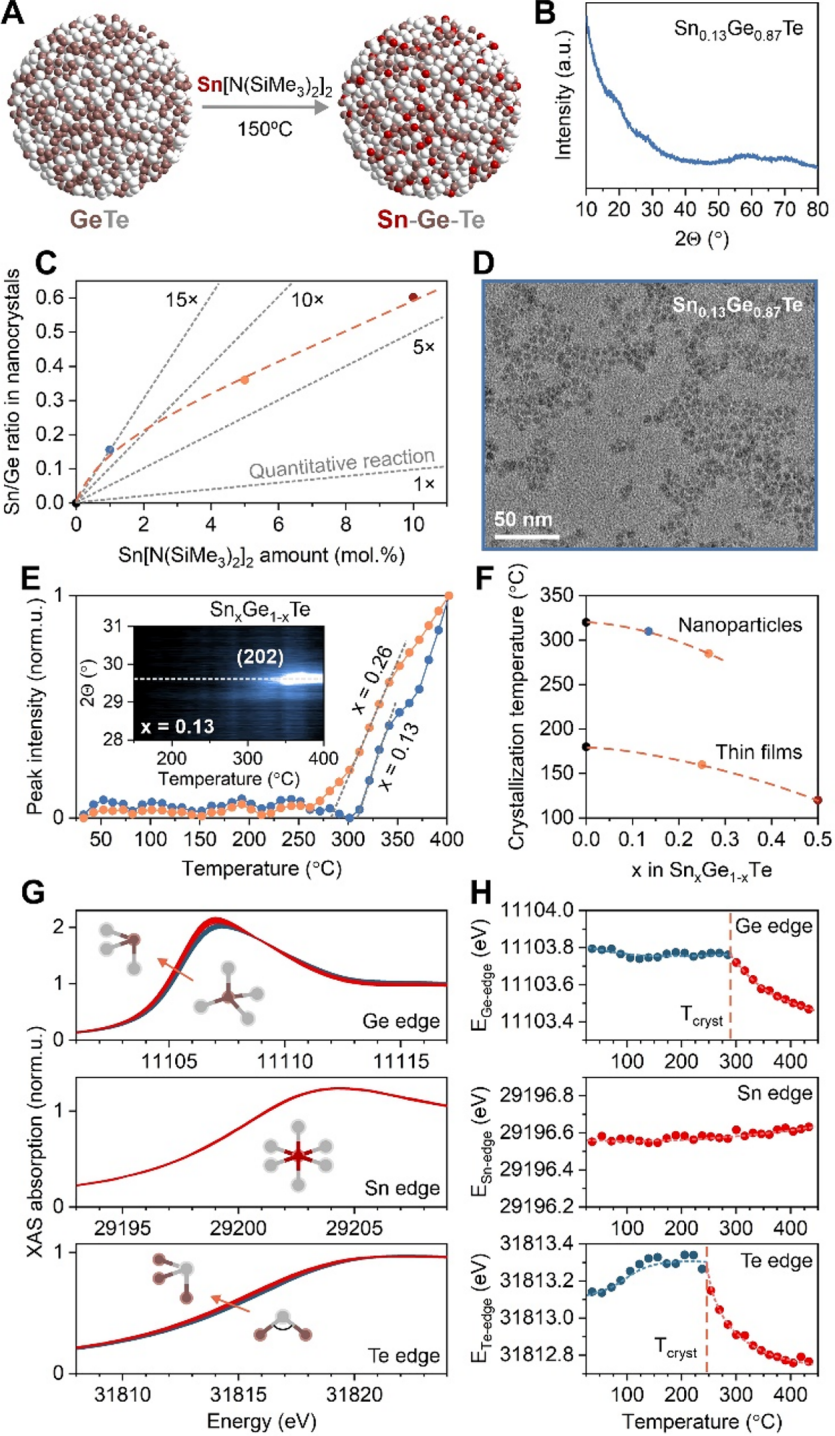
Amorphous Sn–Ge–Te (SGT) nanoparticles. (A) Synthesis schematics, (B) representative X-ray diffraction pattern, (C) composition control, and (D) representative TEM image of 3.5 nm amorphous Sn–Ge–Te nanoparticles. Gray dashed lines in (C) indicate a factor by which Sn[N(SiMe_3_)_2_]_2_ reactivity is higher with respect to GeI_2_. (E) Intensity profiles of the (202) Bragg reflection for amorphous Sn–Ge–Te nanoparticles, extracted from high-temperature XRD measurements (inset E). (F) Composition-dependent crystallization temperature, *T*_cryst_, for Sn–Ge–Te nanoparticles and bulk thin films, derived from line profiles in (E) and from previous literature.^21,40^ (G) X-ray absorption near-edge spectra (XANES) of amorphous Sn_0.15_Ge_0.85_Te nanoparticles and (H) energies of Ge, Sn, and Te K-edges, calculated as the middle of the absorption steps in (G). Insets G are schematics of typical structure units for the amorphous and crystalline Sn–Ge–Te material.

To assess the potential of Sn–Ge–Te QDs for phase-change applications, we must obtain and characterize amorphous Sn–Ge–Te nanoparticles. However, tuning the phase of colloidal nanoparticles is nontrivial and hardly ever reported.^23,41,42^ A direct reaction between metal iodides and a Te precursor yields crystalline Sn–Ge–Te QDs because the high temperature of the synthesis and relatively long reaction times induce crystallization of QD products. We therefore employ a two-step procedure to synthesize amorphous Sn–Ge–Te nanoparticles. First, we prepare amorphous GeTe nanoparticles, following our previous paper.^20^ Afterward, the reaction flask is set to a lower temperature, where we inject a highly reactive Sn-amide precursor, Sn[N(SiMe_3_)_2_]_2_. Figure 3A illustrates the reaction scheme. We obtain Sn–Ge–Te nanoparticles with the amorphous structure (Figure 3B), and its composition can be precisely tuned by the amount of Sn precursor (Figure 3C). Size and narrow size distribution of Sn–Ge–Te nanoparticles are similar to those for GeTe nanoparticles (Figure 3D),^20^ being effectively constant for different amounts of Sn-amide (Figure S6). We therefore conclude that the reaction proceeds through the cation-exchange mechanism.^43^ Sn[N(SiMe_3_)_2_]_2_ is highly reactive in replacing Ge atoms within GeTe nanoparticles: adding just 1 mol % of Sn-amide (relative to GeI_2_ initial amount) leads to around 13% of Ge atoms being replaced by Sn (Figure 3C). Increasing the Sn-amide precursor results in sufficient amounts of Ge-amide byproducts, which start to compete for the surface of Sn–Ge–Te nanoparticles, thus slowing the reactivity of Sn[N(SiMe_3_)_2_]_2_, relative to Ge precursors (Figure 3C).

We then study the structure dynamics of amorphous Sn–Ge–Te nanoparticles using two high-temperature methods: X-ray diffraction (Figure 3E,F) and X-ray absorption spectroscopy (Figure 3G,H). For both measurements, Sn–Ge–Te nanoparticles are heated with comparable constant ramps (5 °C/min for XRD and 6.7 °C/min for XAS). For the X-ray diffraction, we monitor the main (202) Bragg reflection of rhombohedral Sn_*x*_Ge_1–*x*_Te phase and identify the crystallization temperature as the onset of intensity line profiles, in accordance with previous literature (Figure 3E).^20^ We observe proportionally lower crystallization temperatures with increasing Sn content in Sn_*x*_Ge_1–*x*_Te nanoparticles. The same composition trend is known for Sn–Ge–Te thin films,^40^ but for nanoparticles, crystallization temperatures are notably higher and the difference can be as large as 120–150 °C (Figure 3F). The size-dependent crystallization temperature is usually associated with nanoscale effects, such as a fraction of surface atoms which increases the entropy required to commence crystallization.^20^ Alternative explanations rely on the kinetics of crystallization, *e*.*g*., heterogeneous nucleation, viscosity of material due to nanoparticle quantum confinement, or classical nucleation theory.^44^

*In situ* heating X-ray absorption measurements (Figures 3G and S7) provide complementary insight into the structural dynamics of Sn–Ge–Te nanoparticles upon crystallization. By quantifying the shifts in the edge energy (Figure 3H), we can determine relative changes in the oxidation state^45^ and thus deduce the specific roles each element plays during crystallization. Interestingly, we observe that Sn does not change the oxidation state, acting as a stable octahedral center that remains intact well beyond the crystallization point. This is in agreement with previous research on Sn–Ge–Te PCMs, highlighting the important role of Sn doping for structural stability, including the suppression of aging processes and associated resistance drift phenomena.^12,46^ In stark contrast to Sn, Ge atoms decrease the oxidation state upon crystallization, which is indicated by the shift of the XAS edge to lower energies. We relate this observation to the well-documented switch of Ge local environment from tetrahedral to trigonal coordination via the breaking of homopolar Ge–Ge bonds.^47^ While the same trend is valid for Te atoms, we note a slight increase in the edge energy prior to crystallization, signifying that the crystallization process commences via initial preordering of Te atoms, either for the Te sublattice or through the change of Ge–Te–Ge bridge angles.

### Optical Properties of Phase-Change Sn–Ge–Te Thin Films

To produce organic-free and dense thin films, we chemically exchange the native long-chain organic ligands of amorphous Sn–Ge–Te nanoparticles with an inorganic ionic shell using GeI_2_ surface treatment in the two-phase solvent system of hexane and *N*,*N*-dimethylformamide.^20^ FTIR spectroscopy confirms the removal of most fatty acid ligands, showing a 5-times reduced intensity of the C–H stretching modes between 2800 and 3000 cm^–1^, while EDX measurements reveal little changes in the composition of Sn–Ge–Te QDs before and after the ligand exchange (Figure S8). The inorganic-capped Sn–Ge–Te nanoparticles exhibit short-term colloidal stability in *n*-butylamine. This colloidal solution can be spin coated immediately to obtain homogeneous and compact thin films (Figure S8). This is then used for spectroscopic ellipsometry study of Sn–Ge–Te nanoparticle thin films in the amorphous and crystalline state. To obtain the latter, we anneal the amorphous thin film to 300 °C and confirm crystallinity using X-ray diffraction.

**Figure 4:**
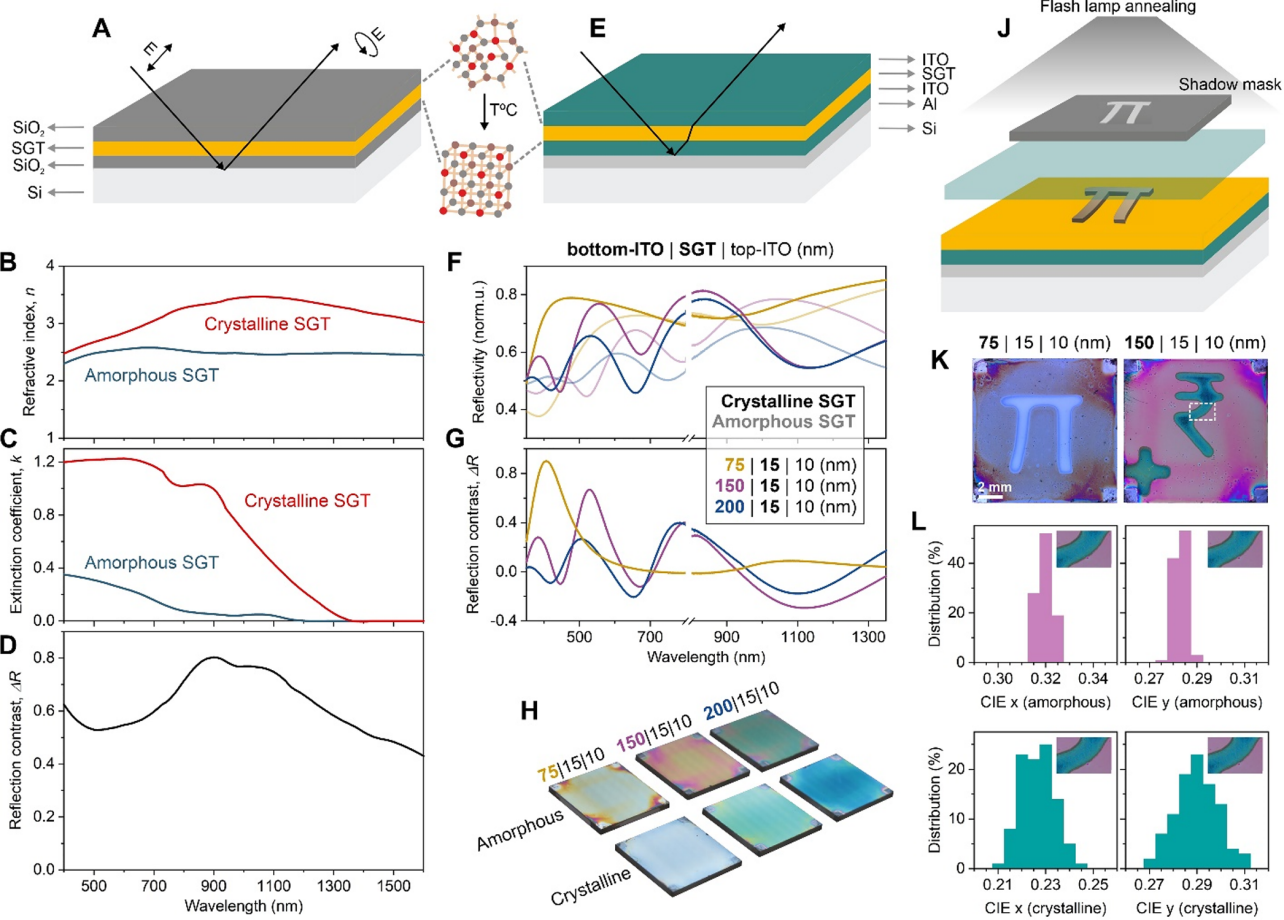
Optical properties of Sn–Ge–Te (SGT) nanoparticles and their application in thin film phase-change optics. (A–D) Ellipsometry measurements: (A) Schematics of the trilayer film used for the ellipsometry measurements. (B) Refractive indices, *n*, and (C) extinction coefficients, *k*, of the film with amorphous and crystalline SGT nanoparticle layers. (D) Reflectivity contrast between the amorphous and crystalline state of the Sn–Ge–Te thin film. (E–H) Demonstration of nonvolatile Sn–Ge–Te phase-change thin films with varying thickness of the bottom ITO layer: (E) Schematics, (F) reflectivity normalized with respect to Al, (G) calculated reflectivity contrast, and (H) photographs of samples with amorphous and crystalline Sn–Ge–Te nanoparticle layers in the reflective stack. (J–L) Nonvolatile static image demonstration, using flash lamp annealing patterning of Sn–Ge–Te thin film stacks: (J) Schematics of experiment, (K) top-view optical microscope images of patterned reflective stacks with varying thickness of the bottom ITO later, (L) CIE color space indices from Sn–Ge–Te amorphous and crystalline regions of a patterned thin film in (K), right panel.

Figure 4A illustrates the stack used for ellipsometry characterization. The samples consist of a Si substrate, followed by 100 nm SiO_2_ and the Sn–Ge–Te thin film, which is capped with SiO_2_ to prevent oxidation. Starting from a Cauchy dispersion model for multilayered thin film samples, we fit the ellipsometry data by a point-by-point algorithm to yield the most precise results (*i*.*e*., the amplitude ratio of the s- and p-polarized components, Ψ(*E*), and the polarization difference between s and p components, Δ(*E*)). For the model fitting, we fix all the refractive indices except for the Sn–Ge–Te thin film and SiO_2_ capping layer to allow for deviations in the deposition process. The model reveals effective refractive indices of the Sn–Ge–Te thin films, which take into account the residual organics and voids. Here, we choose a packing ratio of 0.6 (*i*.*e*., 40% voids in the effective layer), based on our previous study.^48^ Supplementary AFM measurements provide the sample surface roughness for the ellipsometry fits (Figure S8).

Figure 4B and C plot refractive indices (*n*) and extinction coefficients (*k*) of amorphous and crystalline Sn–Ge–Te thin films, which are derived from the fits. The refractive index of Sn–Ge–Te thin films increases upon crystallization and stays larger for the whole measurement spectrum. We observe the largest refractive index change in the technologically important near-IR region (Figure S9). Optical band gaps of amorphous and crystalline Sn–Ge–Te thin films can be estimated from the onsets of extinction coefficient spectra.^48^ We observe narrow band gaps for both Sn–Ge–Te phases (*E*_g,amorph_ = 1.08 eV and *E*_g,cryst_ = 0.92 eV) and a clear ca. 160 meV red shift upon crystallization. Optical band gaps of colloidal Sn–Ge–Te QDs, extracted from the Tauc plots (Figure S10), remain in excellent agreement with the values above. Interestingly, these optical band gaps are wider in comparison to sputtered bulk Sn_*x*_Ge_1–*x*_Te materials,^38,49^ which points to quantum-confinement size effects for Sn–Ge–Te QDs and in nanoparticulate Sn–Ge–Te thin films. Lastly, we calculate the figure of merit (FOM) for Sn–Ge–Te nanoparticle thin films (Figure S9), which is defined as a ratio of refractive index change between amorphous and crystalline state (Δ*n*) to the extinction coefficient of amorphous material (*k*_amorph_) at a specific wavelength.^48^ The FOM is an essential factor to evaluate the potential of material integration into optoelectronic devices.^7^ We observe that Sn–Ge–Te shows high FOM in the near-IR range due to the low extinction coefficient (Figure S9). High FOM values are promising for IR-based optoelectronic devices with minimal damping of optical resonance.^48^

The complex refractive indices can then be used to calculate the reflectivity spectra (*R*) of Sn–Ge–Te thin films in the amorphous and crystalline phase with the following relationship:^50^
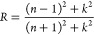


Figure 4D plots the relative reflection contrast, Δ*R*, defined as
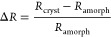


We observe a significant optical contrast for Sn–Ge–Te nanoparticle thin films in both the visible and the near-IR region. A similar trend is known for other phase-change materials (e.g., GeTe and Ge_2_Sb_2_Te_5_), for which an increase in reflectivity upon crystallization is associated with the change in density, confirmed by the Causius–Mossotti law.^35^ In agreement with our results, a recent study on sputtered Sn–Ge–Te thin films reports an increase in reflectivity when switched to the crystalline phase via laser pulses.^11^ The stark reflectivity contrast (Figure 4D) in the visible spectrum is of particular interest for reflective display applications. Here, an amorphous-to-crystalline phase change induces a shift of the resonance wavelength of the reflected light traveling though the multilayered thin film stack, tunable through the thicknesses and material of the individual layers.^51^

### Nonvolatile Reflective Image

Using this distinct reflectivity contrast in our Sn–Ge–Te thin films, we fabricate a simple reflective stack with the Sn–Ge–Te colloids as a memory layer. We do this by incorporating the ligand-exchanged nanoparticle thin film between two indium tin oxide (ITO) layers and on an aluminum (Al) bottommost mirror layer (Figure 4E). To modify the reflection spectra, we adjust thicknesses of the Sn–Ge–Te thin film and the underlying ITO layer. Figure 4F and G show reflectivity spectra and contrast of a stack, in which the ITO thickness varies between 75 and 200 nm (Sn–Ge–Te layer is 15 nm thick). We observe a clear shift in the positions of the spectral maxima before and after annealing of the reflective stack, as the Sn–Ge–Te layer switches between its amorphous and crystalline phase. The color change, induced by the refractive index modulation upon a phase-change of the Sn–Ge–Te layer, is extremely distinct and visible by the naked eye (Figure 4H). Similar effects are observed when tuning the thickness of the Sn–Ge–Te thin film while fixing the underlying ITO layer (Figure S11).

The overall color modulation of the multilayered stack arises due to Fabry–Perot interference as the underlying ITO layer acts like an optical cavity in the phase-change reflective stack.^52^ We also observe strong spectral modulation in the near-IR regime (Figure 4F). Similar effects have already been shown for sputtered thin films, where switching is performed using conductive AFM allowing complete customizable image and pattern creation.^5^ Our results present the next step in this direction, by using liquid-phase processing to achieve faster and improved scaling processes.

Flash lamp annealing (FLA) represents a suitable high-throughput patterning technique, using a high-power xenon lamp to sinter and anneal thin films in a few milliseconds.^53^ Through the predesigned shadow masks, we can crystallize the Sn–Ge–Te reflective stack in the exposed areas. This can be done for variable combinations of layer thicknesses, inducing a vivid color change between amorphous and crystalline Sn–Ge–Te areas (Figure 4K). Thermal simulation of the FLA patterning conditions (Figure S12) shows that the surface temperature of the Sn–Ge–Te thin film rises well beyond the crystallization point (close to 700 °C), while the underlying ITO|Al layers remain below the melting point of Al.

We can quantify the quality of phase-change reflective stacks by extracting CIE color space indices for amorphous and crystalline Sn–Ge–Te areas of optical microscope images (Figure 4L). While observing a clear shift in both indices, we note that the amorphous thin films exhibit notably better color purity (*e*.*g*., narrower distribution of CIE indices). We relate this fact to the high quality and low roughness of the initial amorphous Sn–Ge–Te spin-coated thin films. In contrast, the crystalline area of the reflective stack has a broader distribution of CIE indices (Figure 4L, bottom panels). To investigate the reason behind this, we study the FLA patterned reflective stack under the SEM (Figure S13). In the annealed region, the sample crystallizes in a heterogeneous manner, displaying growth-dominated crystallization. We attribute it to slower crystallization kinetics and clustering of phase-change material, due to the void density in the Sn–Ge–Te nanoparticle layer. Despite this, the nonvolatile reflective images show a noticeable change at the amorphous–crystalline boundary region, observed via electron microscopy (Figure S13) or with the naked eye (Figure 4K). This leads us to believe that further optimization to realize denser phase-change thin films can enhance the reflection intensity and color purity by reducing scattering and extinction effects caused by the voids.

### Electro-Optical Switching

**Figure 5:**
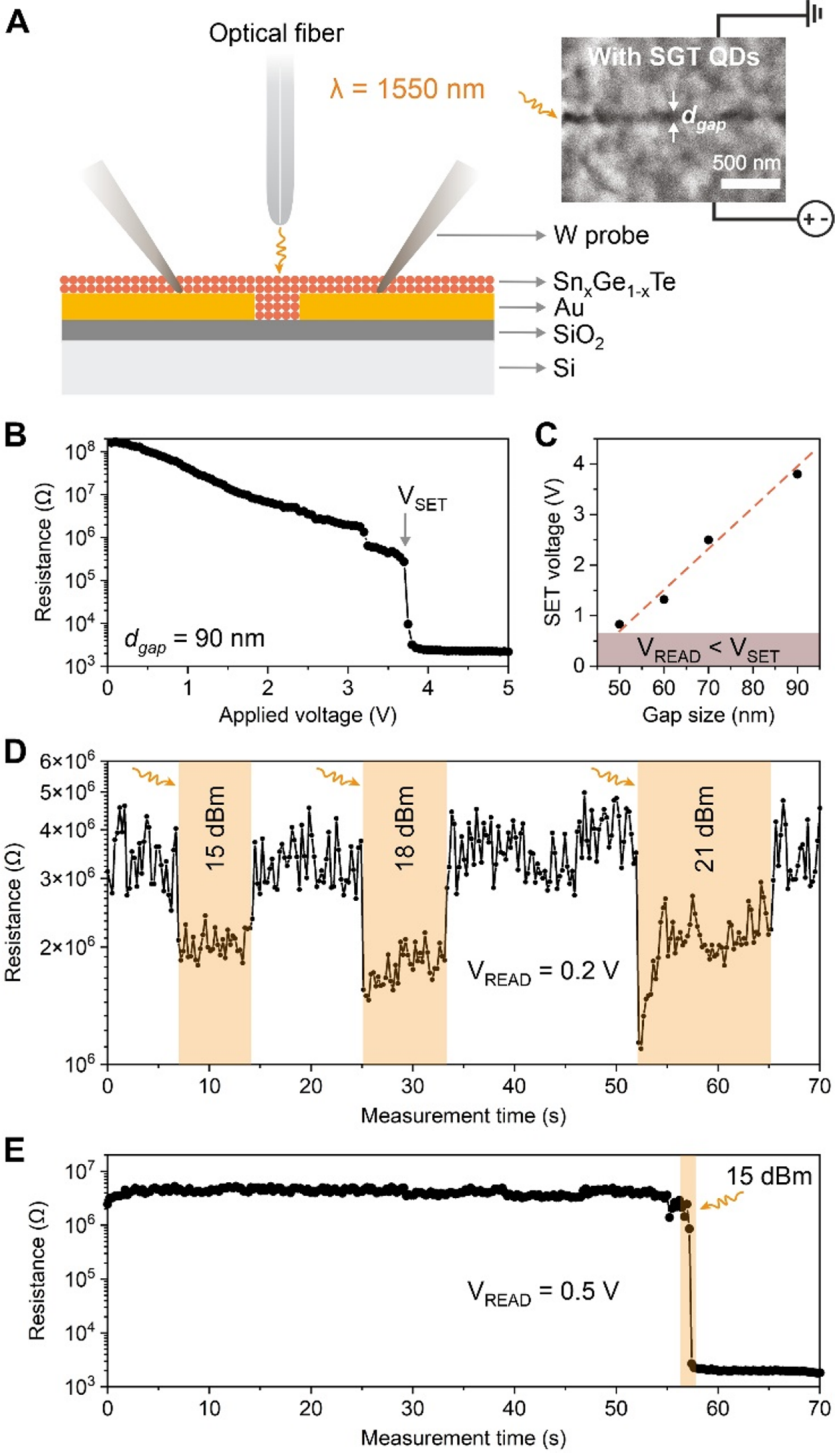
Electro-optical phase-change device with Sn–Ge–Te (SGT) nanoparticles. (A) Schematic of the device architecture and measurement settings. The inset shows the SEM image of Sn–Ge–Te nanoparticles filled into the gap between two Au electrodes in a planar configuration. (B) *R*–*V* characteristics of a Sn–Ge–Te device with a gap size, *d*_gap_, of 90 nm and (C) SET voltages for devices with varying gap size. (D) Volatile electrical response of an amorphous Sn–Ge–Te thin film device under an increasing laser power of 15, 18, and 21 dBm and a read voltage of 0.2 V. (F) Demonstration of the nonvolatile electro-optical switching for the 80 nm wide Sn–Ge–Te device, using a laser power of 15 dBm and a read voltage of 0.5 V.

Since our ellipsometry results point to the phase-change functionality of Sn–Ge–Te thin films in the near-IR, we build a device that can be probed both electrically and electro-optically (Figure 5A). For the latter, we choose an IR laser at the telecom wavelength of 1550 nm as an optical source. Our phase-change devices consist of 100 nm thick Au electrode pairs on a 100 nm SiO_2_|Si substrate. The gap size between the electrodes, *d*_gap_, varies from 50 to 100 nm (Figure S14). The planar configuration of such devices makes it easy to successfully fill these device gaps with Sn–Ge–Te colloids, using a spin-coating deposition method. For electrical characterization, we contact the Au electrodes with tungsten needle probes, while an optic fiber directs the IR laser onto the gap from above (Figure 5A).

Figure 5B shows a typical electrical resistance vs voltage sweep of a Sn–Ge–Te device with a gap size *d*_gap_ = 90 nm. The nonvolatile switch to the low-resistivity SET state happens at 3.6 V. This SET voltage can be lowered by decreasing the gap size (Figure S15); however, 0.8 V is still required to switch the device with *d*_gap_ = 50 nm (Figure 5C). A high SET voltage is unfavorable for device applications since it increases power consumption and can reduce device cyclability by accelerating material degradation.^3^

We therefore introduce the IR laser as an additional high-density energy source to heat a QD thin film locally and thus to minimize the electrical switching voltage. By applying 0.2 V and illuminating the gap with IR light, we observe only a volatile interaction between the laser and Sn-GeTe QDs (Figure 5D). Increasing the optical power leads to proportionally lower resistance of the Sn–Ge–Te thin film, which can be associated with a photoconductivity effect of narrow-band semiconductors, such as ternary tellurides.^34^ Sn–Ge–Te QDs return to their original state when the laser is switched off (Figure 5D). In contrast, increasing the source voltage to 0.5 V facilitates a nonvolatile switching to the low-resistance crystalline Sn–Ge–Te phase as soon as the IR laser is turned on (Figure 5E). Without the laser, however, Sn–Ge–Te devices cannot be switched by source voltages of 0.1–0.5 V (Figure S16), highlighting the importance of the IR laser in this configuration and proving the nature of the light-induced switching.

The electrical resistance measurements demonstrate an excellent resistivity contrast of >10^3^ for our Sn–Ge–Te devices upon crystallization SET (Figure 5E) as well as amorphization RESET switching (Figure S17). The temperature-dependent resistivity measurement (Figure S18) shows an even higher RESET–SET ratio between the amorphous and crystalline state of Sn–Ge–Te thin films, on the order of 10^5^. This alone renders our liquid-fabricated phase-change devices as highly promising candidates for phase-change memory applications. Furthermore, in combination with electro-optical switching at low electrical voltages of 0.5 V, it provides further encouragement for the development of ultra-low-power phase-change devices, prepared via inexpensive and high-throughput non-vacuum deposition of colloidal inks.

## Conclusions

In this work we present a facile and generalizable hot injection approach to prepare a library of ternary telluride QDs (M–Ge–Te, where M is Sn, Bi, Pb, In, Co, and Ag) as promising candidates for phase-change memory applications. We focus on phase-change Sn–Ge–Te nanomaterials with the aim to combine the advantages of Sn-alloying effects (*i*.*e*., suppressed aging and faster speed) and size-dependent crystallization in sub-10 nm QDs. We demonstrate full control for Sn–Ge–Te QDs, reaching a wide range of compositions (*x* = 0–0.7 in the Sn_*x*_Ge_1–*x*_Te) with ultrasmall sizes and uniform size distributions. In a similar, two-step synthetic protocol, we prepare amorphous Sn–Ge–Te nanoparticles with a tunable fraction of Sn atoms.

We continue to thoroughly characterize the structure of our crystalline and amorphous Sn–Ge–Te colloids. Using X-ray methods, we quantify a rhombohedral lattice of crystalline Sn–Ge–Te QDs and study the high-temperature structure dynamics of amorphous Sn–Ge–Te nanoparticles. We relate our findings to the phase-change memory technology, discovering for example a direct correlation between rhombohedral distortion of Sn–Ge–Te and crystallization temperature. Through *in situ* heating X-ray diffraction measurements, we report a decrease in the crystallization temperature for increasing Sn concentration. Furthermore, we observe a large crystallization temperature offset between the bulk and nanomaterials. X-ray absorption spectroscopy aids the discovery of the element-specific role of Ge, Sn, and Te upon crystallization of Sn–Ge–Te nanoparticles, highlighting how Sn atoms act as stabilization centers during crystallization, which in turn reduces resistance drift.

Finally, we show the potential of Sn–Ge–Te colloids in nonvolatile phase-change applications and design several thin film structures, in which the Sn–Ge–Te memory layer consists of inorganic-capped Sn–Ge–Te QDs. Spectroscopic ellipsometry measurements show distinctly different refractive indices and extinction coefficients for the amorphous and crystalline Sn–Ge–Te phase. Using this stark reflectivity contrast, we demonstrate a nonvolatile reflective image on a multicolor Al|ITO|Sn–Ge–Te|ITO reflective stack, patterning it with a flash lamp annealing. Furthermore, we incorporate Sn–Ge–Te QDs into a planar device and show the ability to electro-optically switch the material in the near-IR region at low switching voltages.

Future work may include the waveguide integration of phase-change Sn–Ge–Te QDs and phase-change optical devices, such as near-IR metalenses, tunable filters, beam steerers, or photonic-in-memory computing using wavelength multiplexing.^54^ Taking phase-change optical switches as an example, the signal-light propagation can be manipulated through the changes in refractive indices of the PCM by tuning the phase with a pump laser. The advantages of using a PCM-based optical switch are low power requirement, high ON/OFF ratio, and fast response time. We show that Sn–Ge–Te QDs bring additional benefits, such as a modulation at the telecommunication wavelength regions and a cost-effective fabrication of devices. Taken together, this work will enable an inexpensive way for precise control of resonant frequency and better *Q*-factor for telecommunication applications through inducing multistate crystallization and amorphization of QD-based PCM optical switches. Besides, ternary telluride nanomaterials, reported here, are of high demand beyond the phase-change applications, including thermoelectrics, IR-photodetectors, ferroelectrics, and energy harvesting applications.

## Methods

### Materials

GeI_2_ (99.99%), SnI_2_ (99%), BiI_3_ (99.99%) InBr_3_ (99.99%), AgI (99.9%), tri-*n*-octylphosphine (TOP, 97%), and Te (broken ingots, 99.999%) were purchased from STREM; Sn[N(SiMe_3_)_2_]_2_ (95%) from Gelest; PbI_2_ (99.99%), CoCl_2_ (99%), oleic acid (OA, 90%), chloroform (anh. 99%), ethanol (anh. 99.8%), *n*-butylamine (99.5%), *N*,*N*-dimethylformamide (DMF, anh. 99.8%), hexane (anh. 95%), and toluene (anh. 99.8%) from Sigma-Aldrich; LiN(SiMe_3_)_2_ (95%) from Acros Organics. Oleic acid was dried for 5 h at 110 °C to remove water residues, *n*-butylamine was dried by the freeze–pump–thaw method, and all other chemicals were used as received. All materials and stock solutions were stored inside the air-free N_2_ glovebox, and solvents were protected with molecular sieves.

### Synthesis of Crystalline Sn–Ge–Te Quantum Dots

In an optimized synthesis, anhydrous GeI_2_ (128.5 mg, 0.394 mmol) was dissolved in 9.0 mL of TOP along with SnI_2_ (varying amounts of 0.016–0.230 mmol) in 3.0 mL of TOP at 110 °C on a hot plate in the glovebox. After 20 min of stirring, the orange SnI_2_ and yellow GeI_2_ solutions were left to cool down. The latter was warm-filtered through a 0.2 μm PTFE syringe filter to remove the insoluble residue. The GeI_2_ and SnI_2_ solutions were then mixed and transferred to the dried three-neck flask connected to a vacuum manifold via a Liebig condenser under a N_2_ atmosphere. The mixture of iodides was additionally dried under vacuum at 100 °C for 30 min. Meanwhile, a freshly prepared mixture of TOP:Te (0.8 mL of a 1 M stock solution) and LiN(SiMe_3_)_2_ (0.5 mL of a 1.6 M stock solution) was transferred air-free out of the glovebox and swiftly injected into the three-neck flask containing the GeI_2_ and SnI_2_ reaction mixture at 280 °C and under 1 bar of N_2_. A fast color change from yellow to brown was observed during the first seconds of reaction time, indicating a nucleation and growth of the nanoparticle products. The temperature of the flask dropped by 10–20 °C upon injection. After 30 s, the heating mantle was removed, and the reaction was allowed to continue for another 90 s. Following this, the flask was cooled down using pressurized air and quenched to room temperature using a water bath. The cooled reaction mixture was transferred with a syringe into a vacuum-dried and N_2_-flooded septum-capped container to be transported into the glovebox. The crude mixture was combined with 1.5 mL of dried OA and 10.0 mL of chloroform, shaken rigorously, and left for 5 min. Finally, 25 mL of anhydrous ethanol was added, and the mixture was centrifuged at 6500 rpm for 5 min to separate the nanoparticle precipitates from other reaction byproducts. The Sn–Ge–Te precipitates can be redispersed in chloroform, forming a long-stable solutions of Sn–Ge–Te QDs if stored air-free. Tables S1 and S2 contain reaction conditions for Sn–Ge–Te QDs with tunable composition.

### Synthesis of Amorphous Sn–Ge–Te Nanoparticles

Amorphous Sn–Ge–Te nanoparticles were synthesized by the reaction between amorphous GeTe nanoparticles and a suitable tin precursor, such as Sn[N(SiMe_3_)_2_]_2_. In the first step, we prepared GeTe nanoparticles according to our previously published recipe.^20^ The three-neck flask with as-formed amorphous GeTe nanoparticles was cooled down to room temperature. Meanwhile, a desired amount of Sn[N(SiMe_3_)_2_]_2_ (between 0.004 and 0.04 mmol) was dissolved in 1 mL of TOP in the glovebox. In the second step, the solution of GeTe nanoparticles was brought back to high temperature, and the mixture of Sn[N(SiMe_3_)_2_]_2_ in TOP was swiftly injected at 150 °C. Reaction was allowed for 2 min at 150 °C, and the flask was subsequently quenched using a water bath. Amorphous Sn–Ge–Te nanoparticles were purified and stored air-free and in complete analogy to crystalline Sn–Ge–Te QDs, as described above.

### Synthesis of M–Ge–Te Quantum Dots (M is Bi, Pb, In, Co, and Ag)

Experiments to prepare ternary M–Ge–Te QDs derived from the synthesis of crystalline Sn–Ge–Te QDs by replacing SnI_2_ with BiI_3_, PbI_2_, InBr_3_, AgI, or CoCl_2_ (Table S1). Other reaction conditions, such as injection temperature of 260–280 °C, growth time of 1.5–2 min, and amounts of elemental precursors and solvents were kept similar to the synthesis of crystalline Sn–Ge–Te QDs, described above. We anticipate that other ternary telluride nanoparticles can be prepared in the same fashion and under similar reaction conditions.

### Preparation of Sn–Ge–Te Thin Films

A stable Sn–Ge–Te colloidal solution (1.5 mL, approximately 3 mg/mL) was purified directly prior to thin film fabrication, using 20 mL of ethanol and centrifugation at 3000 rpm for 3 min. The QD precipitates were redispersed in 200 μL of toluene and then mixed with a GeI_2_ solution in DMF (1 mL, 20 mg/mL) and 15 mL of hexane (the amounts of QDs and GeI_2_ were optimized to not alter the Sn–Ge–Te composition, while maximizing the removal of organic ligands). This mixture forms a two-phase system of immiscible solvents, in which Sn–Ge–Te QDs and GeI_2_ ligands transfer to the DMF-rich phase, whereas OA stays in the hexane. After vigorously shaking the mixture for 2 min, the hexane phase was decanted, and 15 mL of fresh hexane was added to the DMF-rich phase. This step, to remove fatty acid ligands, was repeated three more times, and the DMF-based solution of Sn–Ge–Te QDs was eventually precipitated using 20 mL of chloroform and centrifugation at 2000 rpm for 2 min. Finally, inorganic-capped Sn–Ge–Te QDs were redispersed in dried *n*-butylamine, filtered using a 0.2 μm PTFE filter, and deposited immediately afterward. To obtain a 50 nm thin film, 30 μL of Sn–Ge–Te inks was spin coated on 100 nm SiO_2_-grown Si substrates (purchased from Siltronix) under the following deposition conditions: 2000 rpm rotating speed, 1000 rpm/s acceleration, and 30 s dwell time. For thinner films, the ink was diluted proportionally with *n*-butylamine. All-inorganic thin films of Sn–Ge–Te QDs were used for ellipsometry measurement and phase-change optical devices.

### Characterization of M–Ge–Te Quantum Dots and Thin Films

Transmission and scanning transmission electron microscopy (TEM and STEM) were measured using a FEI Talos 200X (200 kV) instrument. Samples were prepared by drop-casting a dilute solution of M–Ge–Te nanoparticles in chloroform on a 200 mesh Cu-supported carbon grid (purchased from Ted Pella). Energy dispersive X-ray (EDX) spectroscopy was taken on FEI Quanta 200 SEM (30 kV) and on FEI Talos 200X (200 kV) microscopes. Analysis of microscopy images was carried out in ImageJ.

X-ray diffraction (XRD) patterns and *in situ* heating XRD measurements were carried out using a Rigaku SmartLab 9 kW system, equipped with a rotating Cu anode and a 2D solid state HyPix-3000 SL detector. For high-temperature XRD, Sn–Ge–Te nanoparticles were mixed with boron nitride (dried at 200 °C under vacuum overnight) and sealed inside a 1.5 mm quartz capillary tube (purchased from Hilgenberg) using epoxy under air-free conditions. The samples were heated at a constant ramp rate of 5 °C/min, scanning a small 2θ region around the (202) Bragg reflection of the rhombohedral GeTe crystal structure.

Absorption spectroscopy of M–Ge–Te colloidal solutions and reflection spectra of Sn–Ge–Te thin film stacks were measured on a Cary 5000 UV–vis–NIR spectrometer. The spectrometer was combined with an integrating sphere using an Al mirror as a reference. Ellipsometry was measured on a rotating analyzer ellipsometer (Woollam VASE) with focusing lenses, which adjust illumination to an approximately 1 × 2 mm spot size (from sample regions where no major defects were present). The data were collected at three different angles between 65° and 75° and from 300 to 1600 nm with a 10 nm step size. Fourier-transform infrared (FTIR) spectroscopy was performed on a Bruker V70 system (InGaAs detector); Sn–Ge–Te QDs were drop-casted on IR-transparent ZnSe windows.

X-ray absorption spectroscopy was measured on the SuperXAS beamline (X10DA) at Paul Scherrer Institute. Sn–Ge–Te nanoparticles were blended with a water-dried boron nitride spacer (approximately 20 wt % of nanoparticles) and loaded into 1 mm thick quartz capillaries (purchased from Hilgenberg). The capillary was capped air-free with epoxy and placed in between a custom built two-lip heater, which was calibrated prior to the measurements. Identical samples of Sn–Ge–Te nanoparticles were measured at the Ge, Sn, and Te K-edges, while heating with a constant ramp rate of 6.7 °C/min. Energy edges were extracted as inflection points of the logistic step functions, fitted to each absorption spectrum.

For the temperature-dependent resistance measurements, interdigitated electrodes (spaced 70 μm apart) were deposited on a glass by evaporating Au via a shadow mask. GeI_2_-capped amorphous Sn–Ge–Te nanoparticle inks were spin-coated on the interdigitated Au electrode structures inside the glovebox. The samples were heated at 2 °C/min on a temperature-controlled hot plate (purchased from Harry Gestigkeit GmbH), and resistance measurements were acquired using a custom-made probe station setup, collecting voltage and current values with a custom-made LabVIEW and Keithley 2400SMU connected to the probe station.

### Nonvolatile Phase-Change Reflective Images

To realize a static image demonstration of the phase-change reflective displays, a sequence of the following layers was deposited on a glass substrate: aluminum mirror (approximately 200 nm), bottom ITO layer, Sn–Ge–Te QD thin film, and top ITO layer. The color of the reflective stack was tuned through the thicknesses of the Sn–Ge–Te thin film and bottom ITO layer. The top ITO layer was 10 nm thick for all samples. The Al layer was thermally evaporated, and ITO layers were deposited by the UNIVEX 450 sputterer from Oerlikon Leybold. The ITO target was obtained from Angstrom Sciences. The following sputtering conditions were used for smooth ITO layers: Ar carrier gas, pressure of 4.5 × 10^–3^ mbar, and power of 160 W.

FLA experiments were carried out using a NovaCentrix, PulseForge 1300 photonic curing system. FLA parameters were optimized to achieve Sn–Ge–Te thin film layer crystallization in the reflective stack configuration. A shadow mask fabricated out of stainless steel was used for patterning the stack. The shadow mask was placed over the reflective stack samples, positioned 10 mm away from a Xe arc lamp with the face of the Sn–Ge–Te film directed toward the incident light. To crystallize the Sn–Ge–Te film, a series of single pulses with a 1500 μs envelope was shot from a 600 V lamp with a total output exposure energy density of 5 J/cm^2^. Sequential pulses were shot until the stack started to exhibit a color change without ablation. Due to the surface nature of the FLA method, it is possible to crystallize the layer without the destruction (such as melting) of the underlying Al layer.

### Electrical and Electro-Optical Measurements

The electrical characterization was performed using the picoampere precision source/measure unit (SMU) B2912A of Keysight. Needle probes of 2 W were used to establish contacts to the Au electrode pads. One Au electrode was grounded, while the other Au electrode was biased with a positive voltage. Due to the symmetry of the device, switching of the nanoparticle thin film was possible in both directions, *i*.*e*., with a positive or a negative voltage. The electro-optical measurements were carried out using a continuous-wave (CW) laser with a wavelength of 1550 nm. The optical signal was amplified and attenuated using the CEDA-C-HG EDFA from Keopsys and the N7764A optical attenuator from Keysight. An optical fiber was placed 10 μm above the nanoparticle-filled gap of the device to ensure direct illumination of the active area (*i*.*e*., free space illumination). An electrical read voltage (*V*_read_) well below the set voltage was applied through the SMU to continuously read out the resistance of the device. During the measurements, a current compliance between 100 and 300 μA was imposed to prevent the device from overheating and potential breakdown.

## Supporting Information Available

Tabulated synthetic conditions for the M–Ge–Te QDs; TEM image of In–Ge–Te; size distribution histograms of M–Ge–Te QDs; composition and size of M–Ge–Te QDs as a function of reaction conditions; X-ray absorption, refractive index change, figure-of-merit, and absorption spectra of Sn–Ge–Te QDs; photos, reflectivity, thermal simulations, and SEM images of Sn–Ge–Te reflective stacks; optical microscopy images, SEM images, *R*–*V* characteristics, and resistance measurements of Sn–Ge–Te phase-change planar devices; high-temperature resistance measurement of Sn–Ge–Te thin film (PDF)
