# Supplementary material for: Colloidal Ternary Telluride Quantum Dots for Tunable Phase Change Optics in the Visible and Near-Infrared
Source: ACS Nano. 2023 Mar 27;17(7):6985–97. doi: 10.1021/acsnano.3c01187 (PMC10100560; doi:10.1021/acsnano.3c01187)
Supplement: Supplementary file 1 — nn3c01187_si_001.pdf [file nn3c01187_si_001.pdf]

Supplementary Information for

Colloidal Ternary Telluride Quantum Dots for Tunable Phase Change Optics in the Visible and Near-Infrared

Dhananjeya Kumaar,[1] Matthias Can,[1] Kevin Portner,[2] Helena Weigand,[3] Olesya Yarema,[4] Simon Wintersteller,[1] Florian Schenk,[1] Darijan Boskovic,[1] Nathan Pharizat,[1] Robin Meinert,[2] Evgeniia Gilshtein,[5] Yaroslav Romanyuk,[5] Artemios Karvounis,[3] Rachel Grange,[3] Alexandros Emboras,[2] Vanessa Wood,[4] Maksym Yarema[1,*]

[1]Chemistry and Materials Design, Institute for Electronics, Department of Information Technology and Electrical Engineering, ETH Zurich, 8092 Zurich, Switzerland

[2]Integrated Systems Laboratory, Department of Information Technology and Electrical Engineering, ETH Zurich, 8092 Zurich, Switzerland

[3]Optical Nanomaterial Group, Institute for Quantum Electronics, Department of Physics, ETH Zurich, 8093 Zurich, Switzerland

[4]Materials and Device Engineering, Institute for Electronics, Department of Information Technology and Electrical Engineering, ETH Zurich, 8092 Zurich, Switzerland

[5]Laboratory for Thin Films and Photovoltaics, Empa – Swiss Federal Laboratories for Materials Science and Technology, 8600 Dübendorf, Switzerland

* Correspondence to: yaremam@ethz.ch

Table S1 | Synthetic conditions for M-Ge-Te nanoparticles via amide-promoted synthesis. Metal iodides were used in all cases, expect for Co, where chloride precursor was taken

#	Material	GeI_2, mmol	M halide, mmol	TOP:Te, mmol	$Li[N(CH_3)_2]$, mmol	T_{inject}, °C	t_{growth}, min	Ge, at.%	M, at.%	Te, at.%
1	Sn-Ge-Te	0.394	0.0197	0.8	0.80	280	1.5	38.48	4.36	57.16
2	Sn-Ge-Te	0.394	0.0197	0.8	0.80	280	1.5	36.62	4.73	58.65
3	Sn-Ge-Te	0.394	0.0394	0.8	0.96	280	1.5	32.86	8.51	58.63
4	Sn-Ge-Te	0.394	0.0788	0.8	0.96	280	1.5	30.98	10.09	58.93
5	Sn-Ge-Te	0.394	0.1182	0.8	0.96	280	1.5	24.49	15.95	59.56
6	Sn-Ge-Te	0.394	0.1970	0.8	1.28	280	1.5	14.72	25.56	59.72
7	Sn-Ge-Te	0.394	0.2758	0.8	1.44	280	1.5	12.85	28.38	58.77
8	Sn-Ge-Te	0.394	0.0591	0.8	0.96	280	1.5	31.00	14.70	54.30
9	Bi-Ge-Te	0.394	0.0079	0.8	0.96	260	1.5	29.01	22.64	48.35
10	Bi-Ge-Te	0.394	0.0197	0.8	0.96	260	1.5	17.43	24.07	58.5
11	Bi-Ge-Te	0.394	0.0394	0.8	0.96	260	1.5	10.17	29.94	59.89
12	Pb-Ge-Te	0.394	0.0048	0.8	0.80	280	1.5	41.46	2.76	55.78
13	Pb-Ge-Te	0.394	0.0120	0.8	0.80	280	1.5	37.65	4.95	57.40
14	Pb-Ge-Te	0.394	0.0180	0.8	0.80	280	1.5	39.03	6.08	54.89
15	Pb-Ge-Te	0.394	0.0311	0.8	0.80	280	1.5	34.32	9.00	56.68
16	In-Ge-Te	0.394	0.0197	0.8	0.80	250	1.5	43.79	4.88	51.33
17	In-Ge-Te	0.394	0.0591	0.8	0.80	250	1.5	39.10	7.93	52.97
18	Co-Ge-Te	0.297	0.0300	0.8	0.96	280	2.0	43.01	13.81	43.18
19	Ag-Ge-Te	0.297	0.0300	0.8	0.96	280	2.0	35.91	6.70	57.39
20	Ag-Ge-Te	0.328	0.3300	1.6	1.60	265	1.0	11.57	41.32	47.11
21	Ag-Ge-Te	0.328	0.5000	1.6	1.60	265	2.0	5.98	51.51	42.50

Table S2 | Synthetic conditions for crystalline Sn-Ge-Te nanoparticles via amide-promoted synthesis

#	GeI_2, mmol	SnI_2, mmol	TOP:Te, mmol	$Li[N(CH_3)_2]$, mmol	T_{inject}, °C	t_{growth}, min	Ge, at.%	Sn, at.%	Te, at.%	Sn content, cat.%
1	0.394	0.0394	0.8	0.64	280	1.5	36.90	6.73	56.37	0.154
2	0.394	0.0394	0.8	0.80	280	1.5	36.44	7.38	56.18	0.168
3	0.394	0.0394	0.8	0.88	280	1.5	29.90	8.53	61.57	0.222
4	0.394	0.0394	0.8	0.96	280	1.5	32.86	8.51	58.63	0.206
5	0.394	0.0394	0.8	1.04	280	1.5	27.16	10.63	62.21	0.281
6	0.394	0.0394	0.8	1.12	280	1.5	26.79	10.87	62.34	0.289
7	0.394	0.0394	0.8	1.28	280	1.5	26.27	10.60	63.13	0.287
8	0.394	0.0394	0.8	1.44	280	1.5	30.25	12.65	57.10	0.295
9	0.394	0.0394	0.8	0.80	260	1.5	23.48	19.29	57.23	0.451
10	0.394	0.0394	0.8	0.80	270	1.5	33.26	8.71	58.03	0.208
11	0.394	0.0394	0.8	1.12	270	1.5	22.98	12.67	64.35	0.355
12	0.394	0.0394	0.8	0.80	290	1.5	34.50	6.15	59.35	0.151
13	0.394	0.0394	0.8	1.12	290	1.5	29.42	9.81	60.77	0.250
14	0.394	0.0394	0.8	1.12	300	1.5	30.64	7.21	62.15	0.190

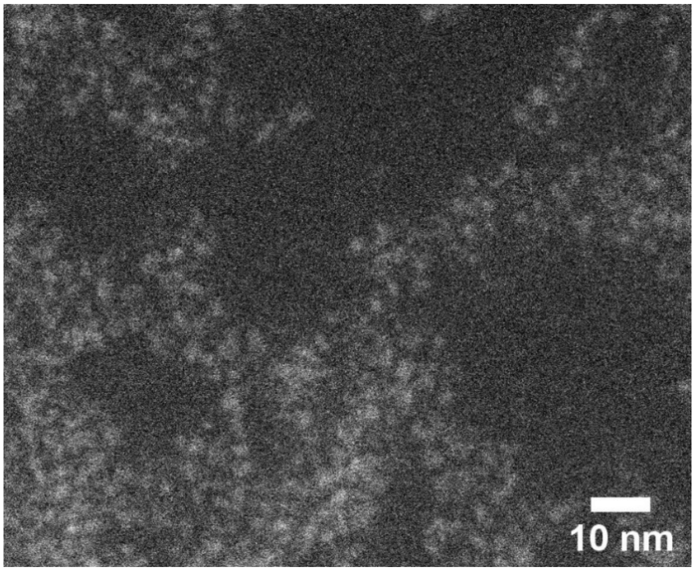

Figure S1 | HAADF STEM image of ultrasmall In-Ge-Te nanoparticles

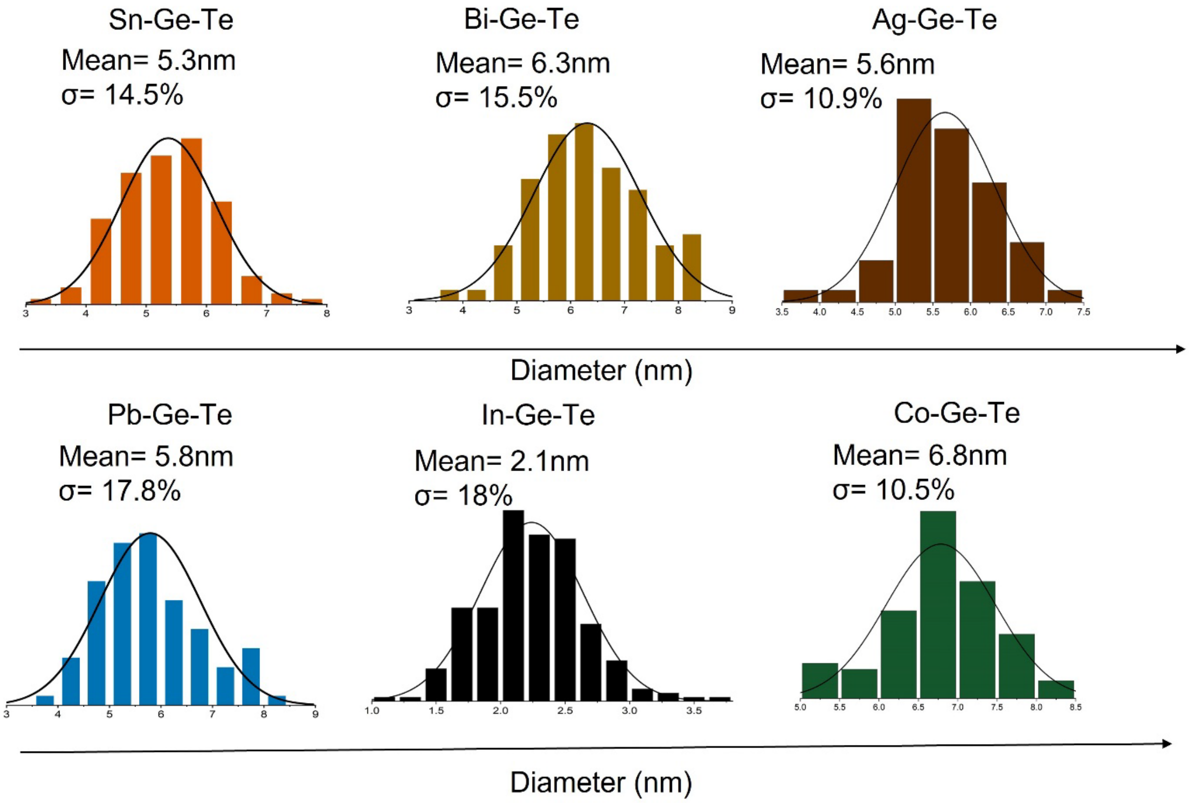

Figure S2 | Size distributions of M-Ge-Te nanoparticles

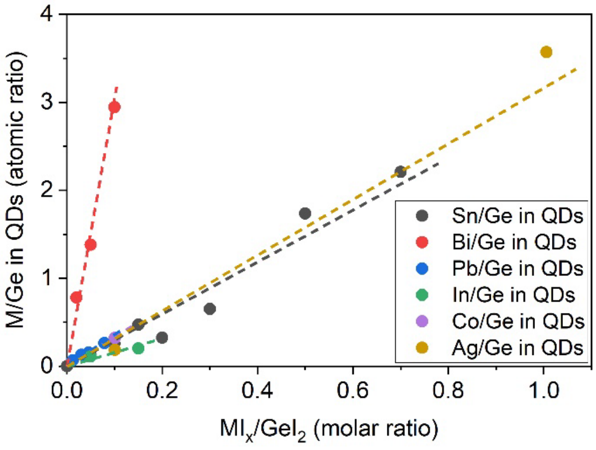

Figure S3 | Composition of M-Ge-Te nanoparticles as a function of precursor ratio

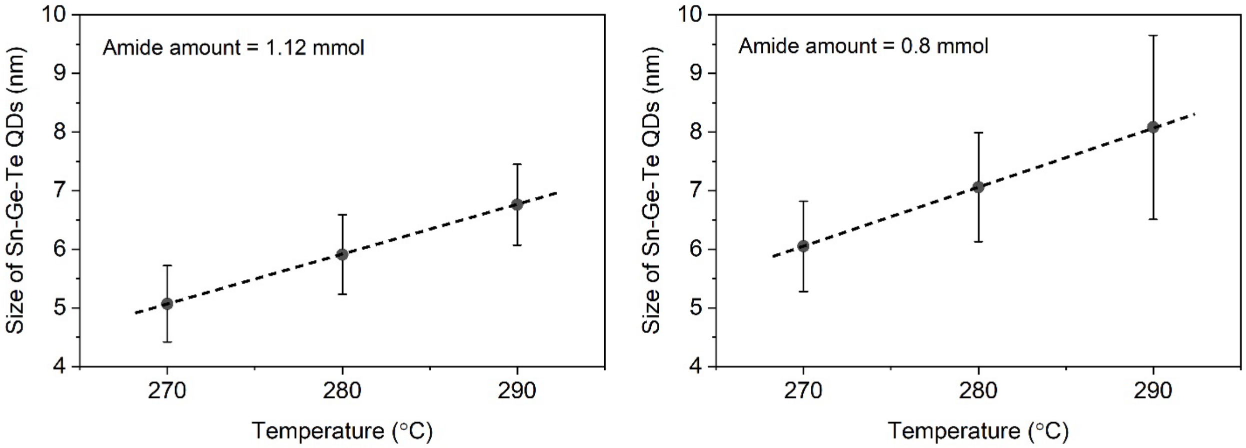

Figure S4 | Size of Sn-Ge-Te nanoparticles, synthesized at different injection temperatures

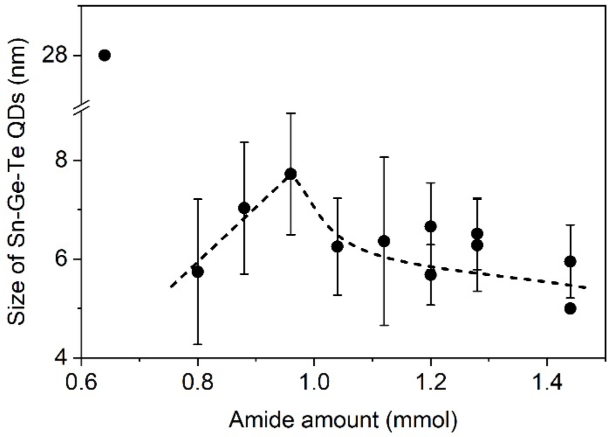

Figure S5 | Size of Sn-Ge-Te nanoparticles for a series of syntheses with variable amount of lithium amide (injection temperature is 280°C)

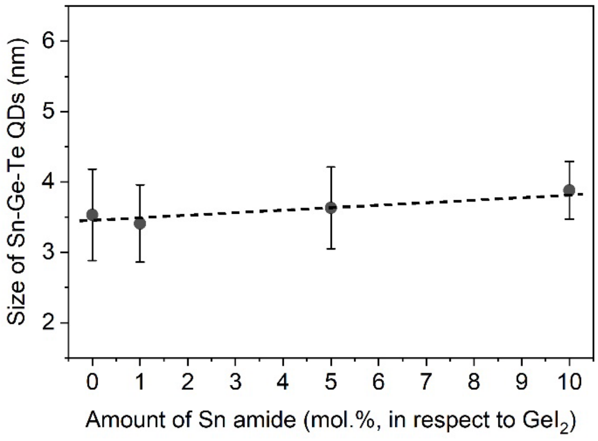

Figure S6 | Size of amorphous Sn-Ge-Te nanoparticles from injecting different amounts of Sn amide

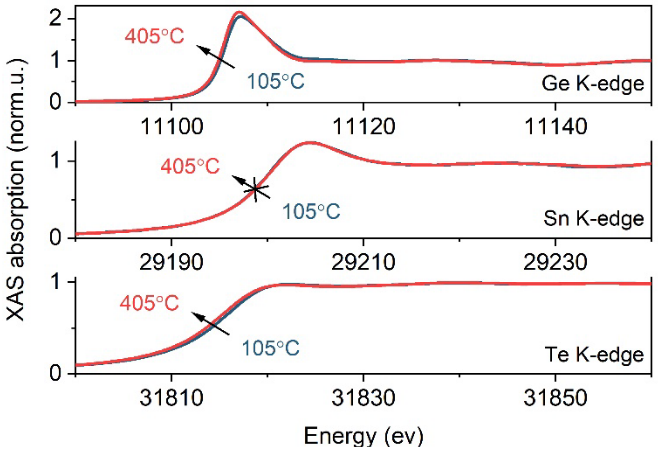

Figure S7 | Full EXAFS spectra of Sn-Ge-Te nanoparticles prior crystallization (at 105°C) and after it (at 400°C). Clear shifts are visible for the Ge K-edge (top) and Te K-edge (bottom); no observable shift of the XAS spectrum for the Sn K-edge (middle).

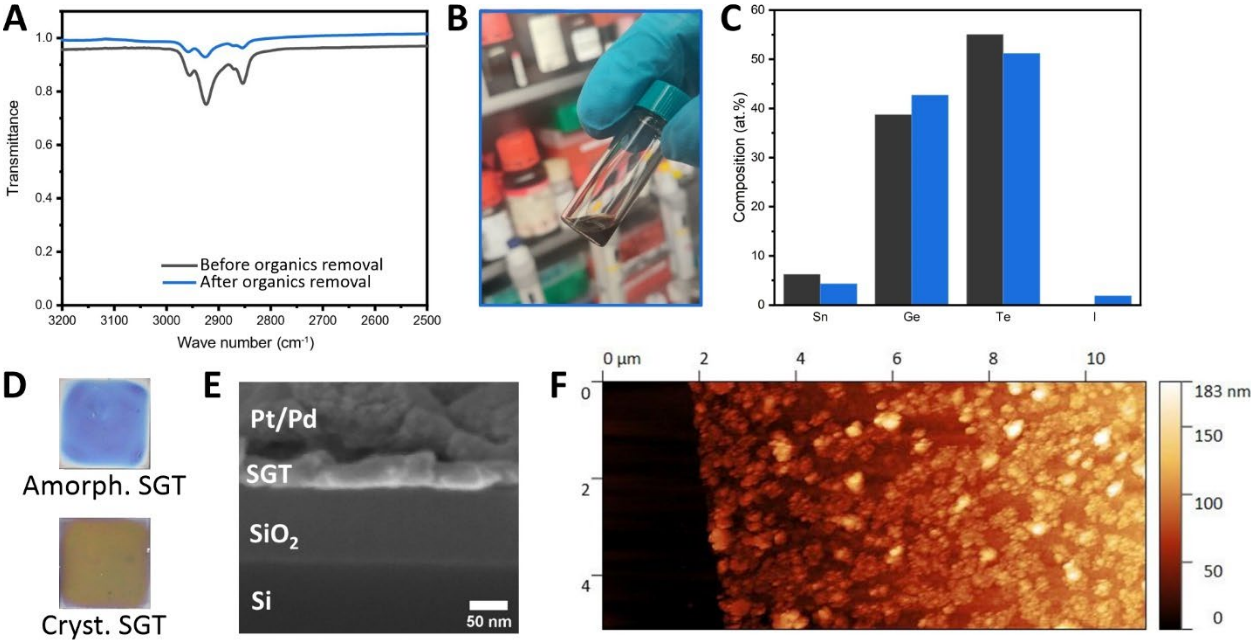

Figure S8 | Ligand exchange with GeI_2 using phase transfer method and thin film fabrication of Sn-Ge-Te quantum dots. Original organic ligands of the Sn-Ge-Te nanoparticles were replaced with GeI_2, which is proven by FTIR spectroscopy (A). The inorganic-capped nanoparticles remained colloidally stable for approx. 1h in n-butylamine (B) and we noted that elemental composition of Sn-Ge-Te colloids is little affected by the ligand exchange (C). The Sn-Ge-Te thin film was prepared by a spin-coating (D) and the amorphous Sn-Ge-Te thin film was annealed to 300°C to form a crystalline film. The SEM micrograph shown in (E) and the AFM image shown in (F) are for the annealed SGT thin film.

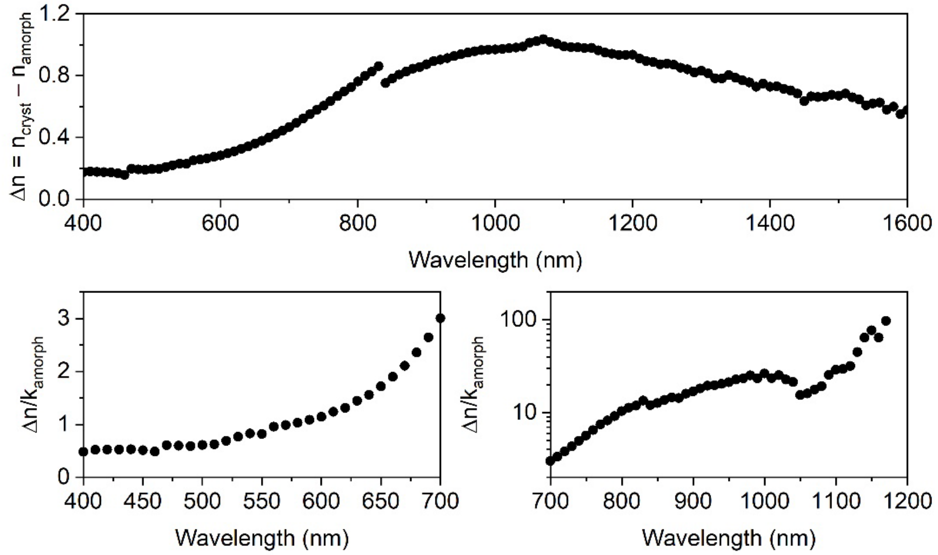

Figure S9 | Refractive index change, Δn, and figure-of-merit (FOM; $\Delta n/k_{amorph}$) spectra for the ligand-exchanged Sn-Ge-Te thin film

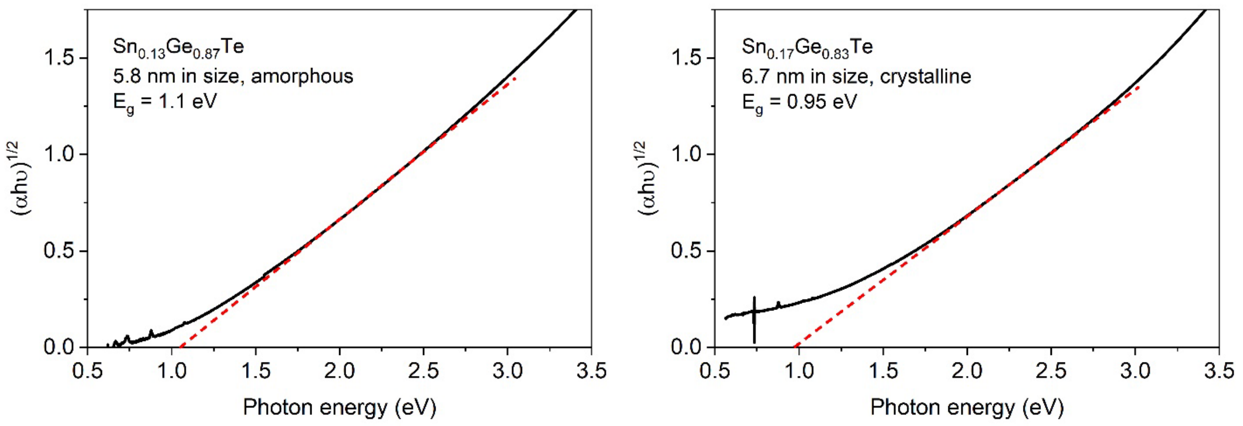

Figure S10 | Absorption spectra of amorphous and crystalline Sn-Ge-Te colloids, presented as Tauc-type plots. The band gaps can be estimated by extrapolating a linear part of the spectrum to 0 and these values stay in good agreement with the band gap estimates for the thin films (as onsets of extinction coefficient spectra; see main text). Although no photoluminescence signal was observed for these samples, we see a band gap widening for Sn-Ge-Te nanoparticles, hinting towards quantum confinement size effects for these colloids.

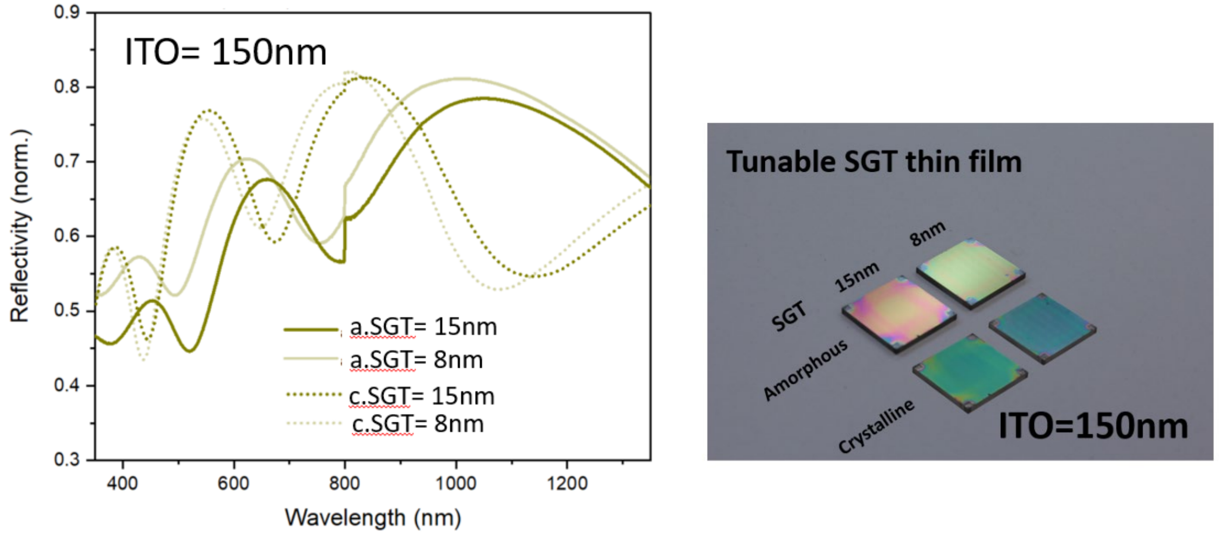

Figure S11 | Demonstration of non-volatile Sn-Ge-Te phase-change thin films with varying thicknesses of the Sn-Ge-Te memory layer. Reflectivity normalized with respect to aluminum and a photograph of samples with the amorphous and crystalline Sn-Ge-Te nanoparticle layer in the reflective stack.

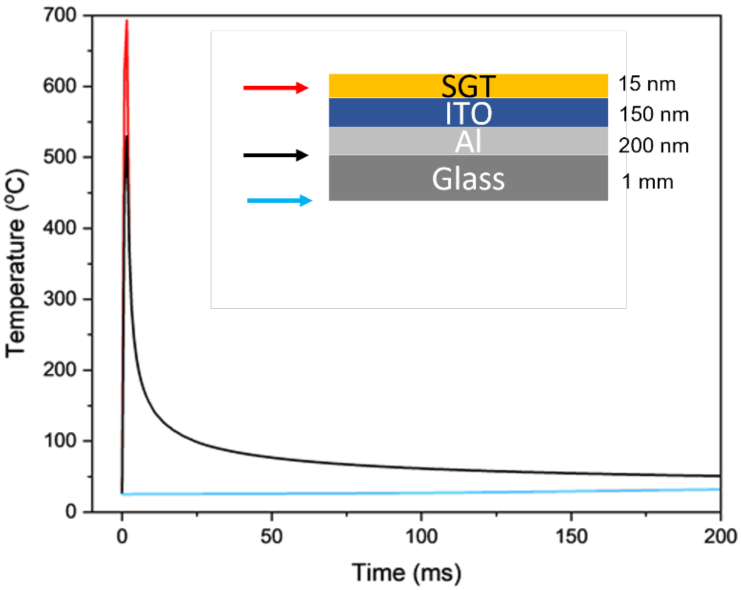

Figure S12 | Thermal simulations of Flash lamp annealing (FLA) conditions. The SimPulse tool was used to estimate the temperatures reached on the surface of the layer and at the interfaces of the layers. It couples a transient 1-D heat conduction model to temperature-dependent thermal and optical material properties. The thermal conductivity of Sn-Ge-Te used here corresponds to the total thermal conductivity of bulk Sn-Ge-Te as an approximation. For the simulations of the temperature profiles, a 15 nm Sn-Ge-Te film on 75/150 nm ITO and 200 nm Al on 1 mm glass substrate were selected as layer stack for the SimPulse tool. Temperature profiles for the used FLA settings described above are demonstrated in the plot shown here. On the surface of the SGT film, an annealing temperature reaching close to 700 °C was estimated. Such a high surface temperature ensures sufficient crystallization of the film. Simultaneously, the Al layer underneath the transparent ITO layer remained below the melting point of the Al thin films (660-690°C).

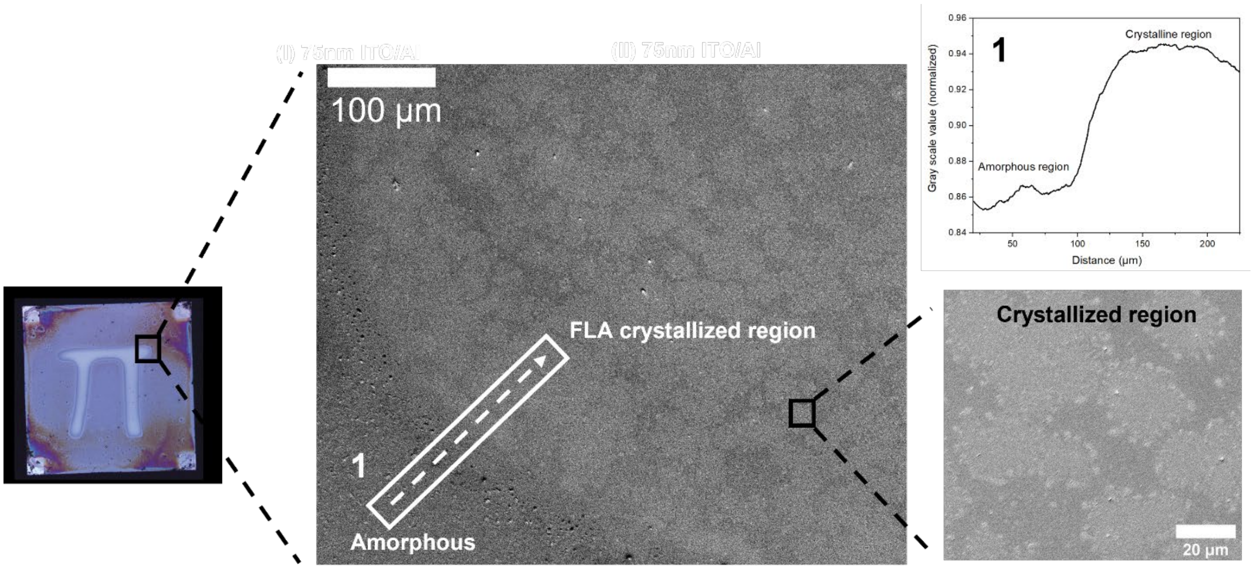

Figure S13 | FLA patterned Sn-Ge-Te reflective stack with 75nm ITO underlayer. Here we observe the crystalline region showing growth-dominated crystallization behavior.

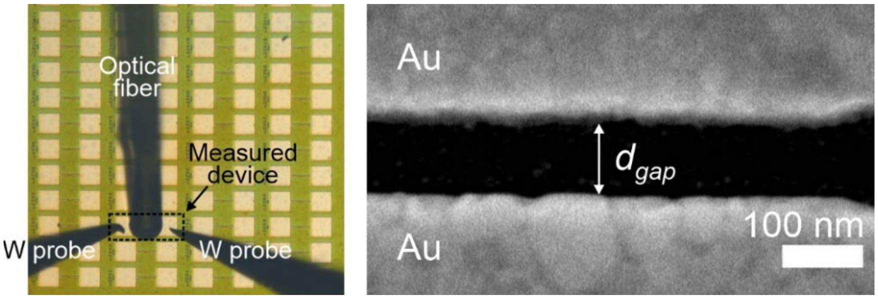

Figure S14 | Optical microscopy image of a chip with Sn-Ge-Te nanocrystal layer on the top (left) and a close-up view on the gap before the Sn-Ge-Te deposition (SEM image, right). Optical microscope image shows an arrays of Au electrode pairs with tungsten electrical probes and an optical fiber on one device. The electrical probes were used to either electrically switch the phase change material by applying a source voltage ($V_{applied}$) or to read out the resistance state by applying a read voltage (V_{read}) while an optical signal was applied through the optical fiber.

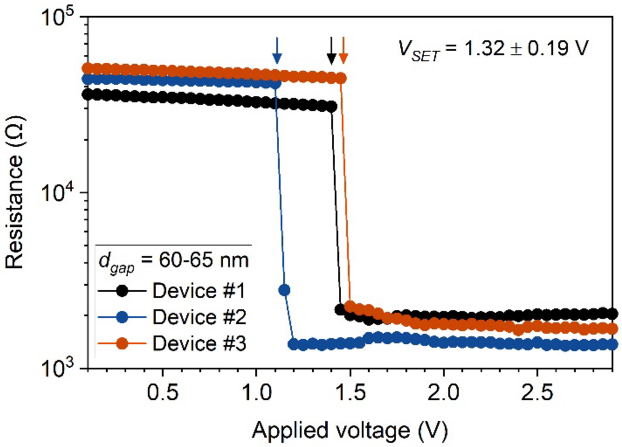

Figure S15 | Resistance-Voltage characteristics of Sn-Ge-Te devices with a gap size of 60-65 nm. The mean SET voltage for these measurements is 1.32 V and the error of experiment is approx. 10-15%. This error comes mainly from the fabrication aspects of the planar devices, such as the innate edge roughness, an offset between the two lithography layers, inhomogeneity of QD thin film, etc. At the same time, the resistance stays similar and do not correlate with the SET voltage values of individual devices, indicating no major problems with contact resistance of our set-up.

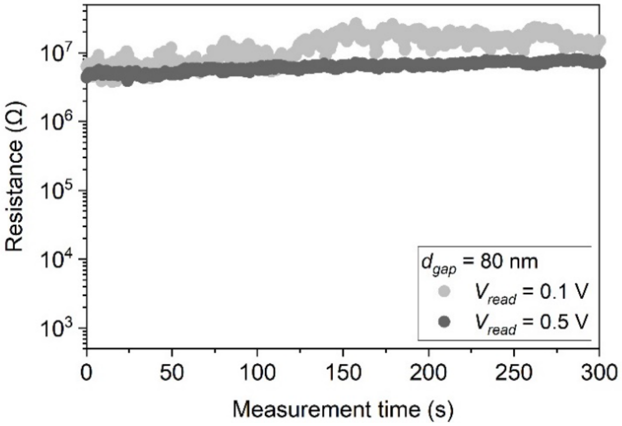

Figure S16 | Resistance of a device with a gap size, d_{gap} = 80 nm, measured at V_{read} = 0.1 V and 0.5 V. No electrical switching event was induced by applying voltages of 0.1-0.5 V.

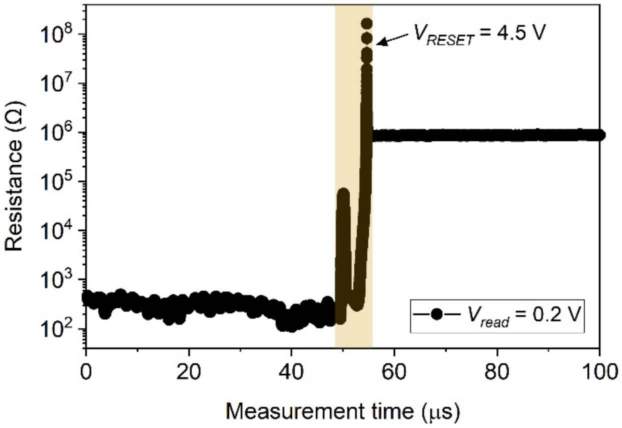

Figure S17 | Resistance of a device before and after a very short high voltage pulse (4.5 V, 200 ns). In the plot shown above, we can electrically RESET our QD-based planar devices, proving the principle that QD-based phase-change switches are indeed reversible. We observe an excellent resistivity contrast upon RESET.

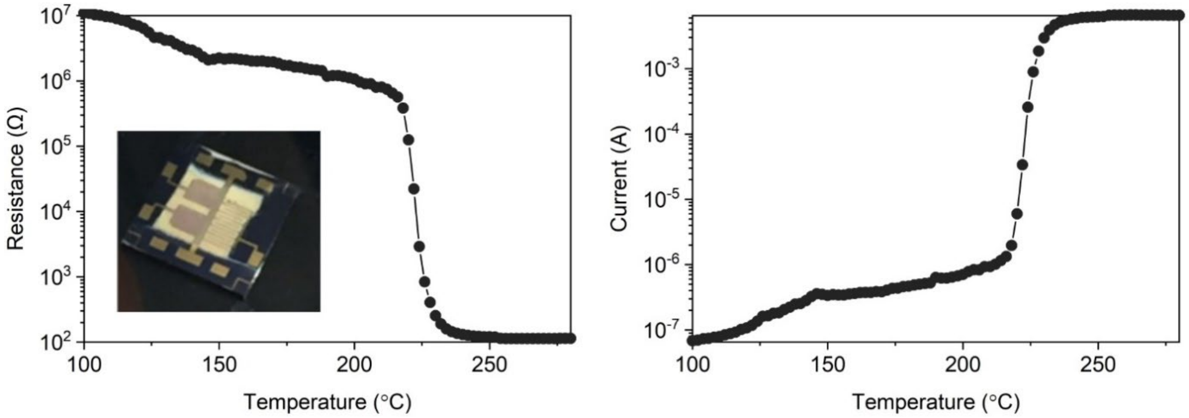

Figure S18 | Resistance and electrical current vs. temperature for the Sn-Ge-Te thin film sample. The Sn-Ge-Te was ligand exchanged with GeI_2 to improve electrical conductivity between the nanoparticles. Resistance was measured in-situ at a temperature ramp of 2°C/min in two-probe mode under constant bias voltage of 0.5 V. A change from the high resistance to low resistance state is observed to take place at 215°C, which is notably higher than bulk Sn-Ge-Te thin films. The experiment was conducted using a custom-made setup in the lab. Inset shows a sample before the measurements.
